# Supplementary material for: Transposon end recognition and excision mechanisms of type I-F CRISPR-associated transposases
Source: Nat Commun. 2026 Aug 24;17:10110. doi: 10.1038/s41467-026-76893-7 (PMC13598128; doi:10.1038/s41467-026-76893-7)
Supplement: Supplementary file 1 [file 41467_2026_76893_MOESM1_ESM.pdf]

Supplementary Information

Transposon end recognition and excision mechanisms of type I-F CRISPR-associated transposases

Mateusz Walter, Giada Finocchio, Seraina Oberli, Iana C. Hammerschmid, George D. Lampe, Julia Karan, Thomas Swartjes, Samuel H. Sternberg, Martin Jinek and Irma Querques

Supplementary Figures

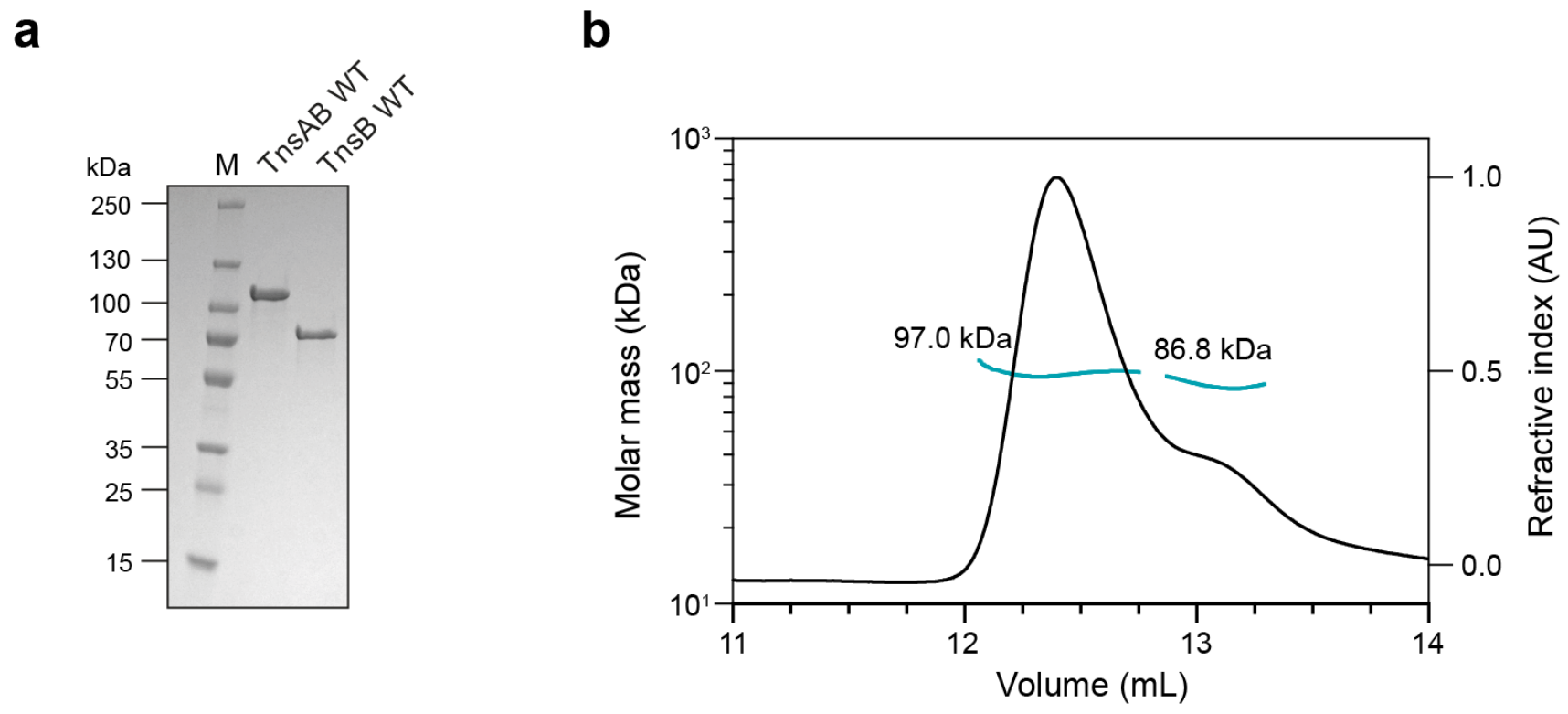

Supplementary Fig. 1 | Purification and characterization of *Pse*TnsAB

(**a**) SDS-PAGE analysis of the purified *Pse*TnsAB and *Pse*TnsB proteins. WT, (wild-type). M, marker. (**b**) Size exclusion chromatography coupled with multi-angle light scattering (SEC-MALS) of purified *Pse*TnsAB. The results align with the theoretical molecular weight of a monomer (97.65 kDa). Source data are provided as a Source Data file.

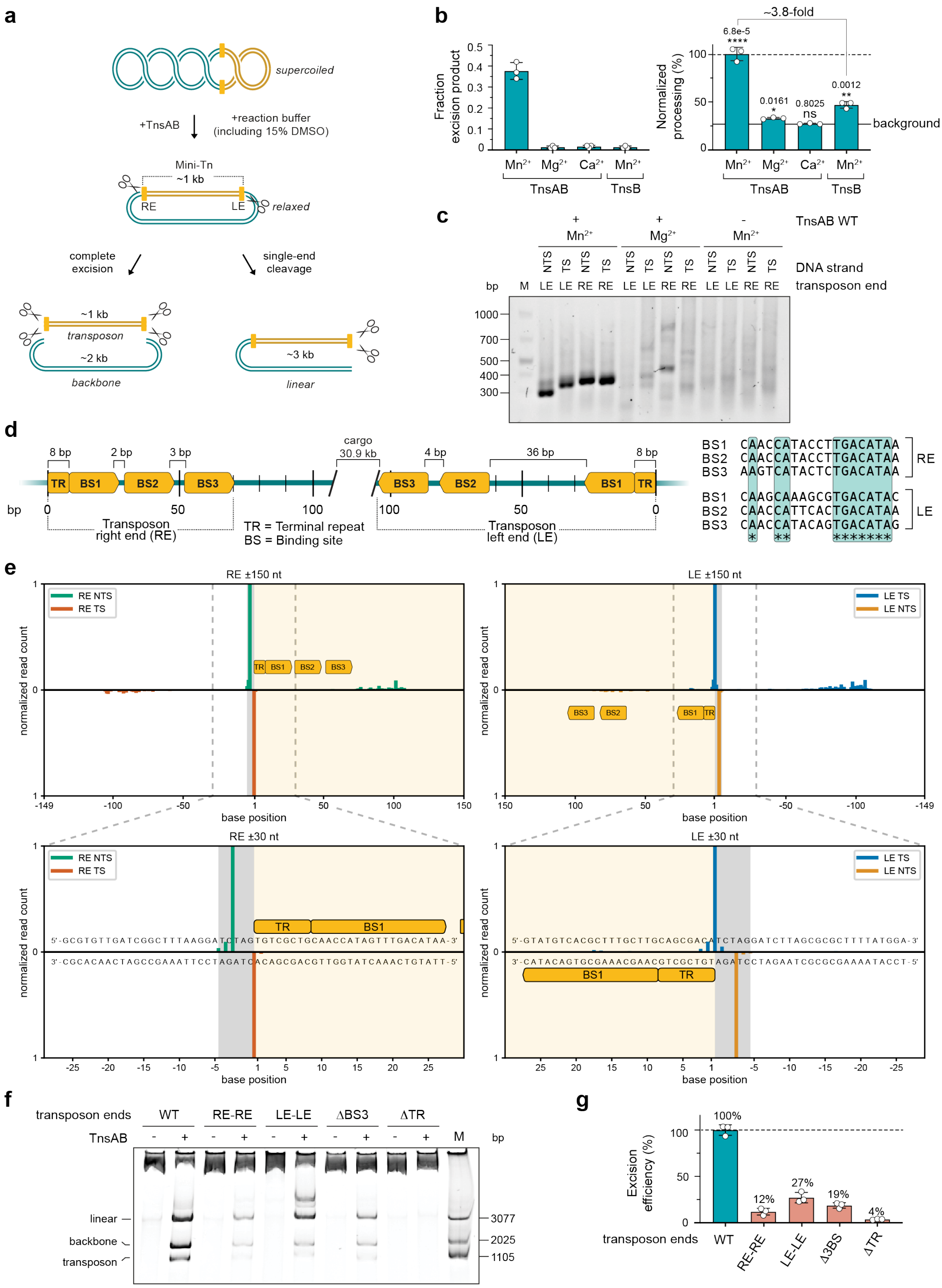

Supplementary Fig. 2 | Reconstitution and analysis of *Pse*TnsAB cleavage activity *in vitro*

(**a**) Schematic of the *in vitro* excision assay. Mini-Tn, mini-transposon. LE/RE, left/right end. (**b**) Quantification of *in vitro* excision from three independent experiments (see **Methods** for details). Relative amounts of DNA species were determined by densitometric analysis. Left: Quantification of excision. Data show mean ± s.d. (n = 3 independent experiments) of plasmid backbone (~2 kb-long

excision product). Right: Quantification of total DNA processing, normalized against WT TnsAB in the presence of Mn^{2+}. Data show mean ± s.d. (n = 3 independent experiments) of all processed DNA species. The solid horizontal line indicates the mean background level of processed plasmid in the no-protein control. Statistical significance was assessed using unpaired two-tailed *t*-tests. P values above each graph indicate comparisons to the no-protein control. Significance is denoted as *P < 0.05; **P < 0.01; ****P < 0.0001; ns, not significant. Exact P values are shown above the asterisks. (**c**) Nested PCR products resolved by agarose gel electrophoresis. TS, transferred strand; NTS, non-transferred strand. (**d**) Left: Arrangement of transposon ends. TR, terminal repeat; BS, binding sites. Minor ticks every 10 bp. Right: Alignment of RE and LE BS sequences, with conserved nucleotides marked by asterisks. (**e**) Cleavage positions inferred from Illumina sequencing. y-axis, normalized read counts per cut site. Bars above/below axis denote top/bottom strand cuts. Transposon end regions are shaded yellow; target site duplications (TSDs) grey. TRs and BSs are highlighted in bright yellow. Windows cover ≥99.999% of reads for each strand/end. (**f**) *In vitro* excision assay with mutant plasmids carrying wild-type (WT), symmetrized, or truncated ends. DNA species are resolved by native PAGE. Representative experiment is shown. ΔBS3, lacking BS3 at both ends; ΔTR, lacking terminal repeats at both ends; M, marker. (**g**) Quantification of *in vitro* excision assay with mutant plasmids. Mean ± s.d. of plasmid backbone (~2 kb-long excision product) normalized to WT (n = 3 independent experiments), as measured by densitometry (see **Methods** for details). Source data are provided as a Source Data file.

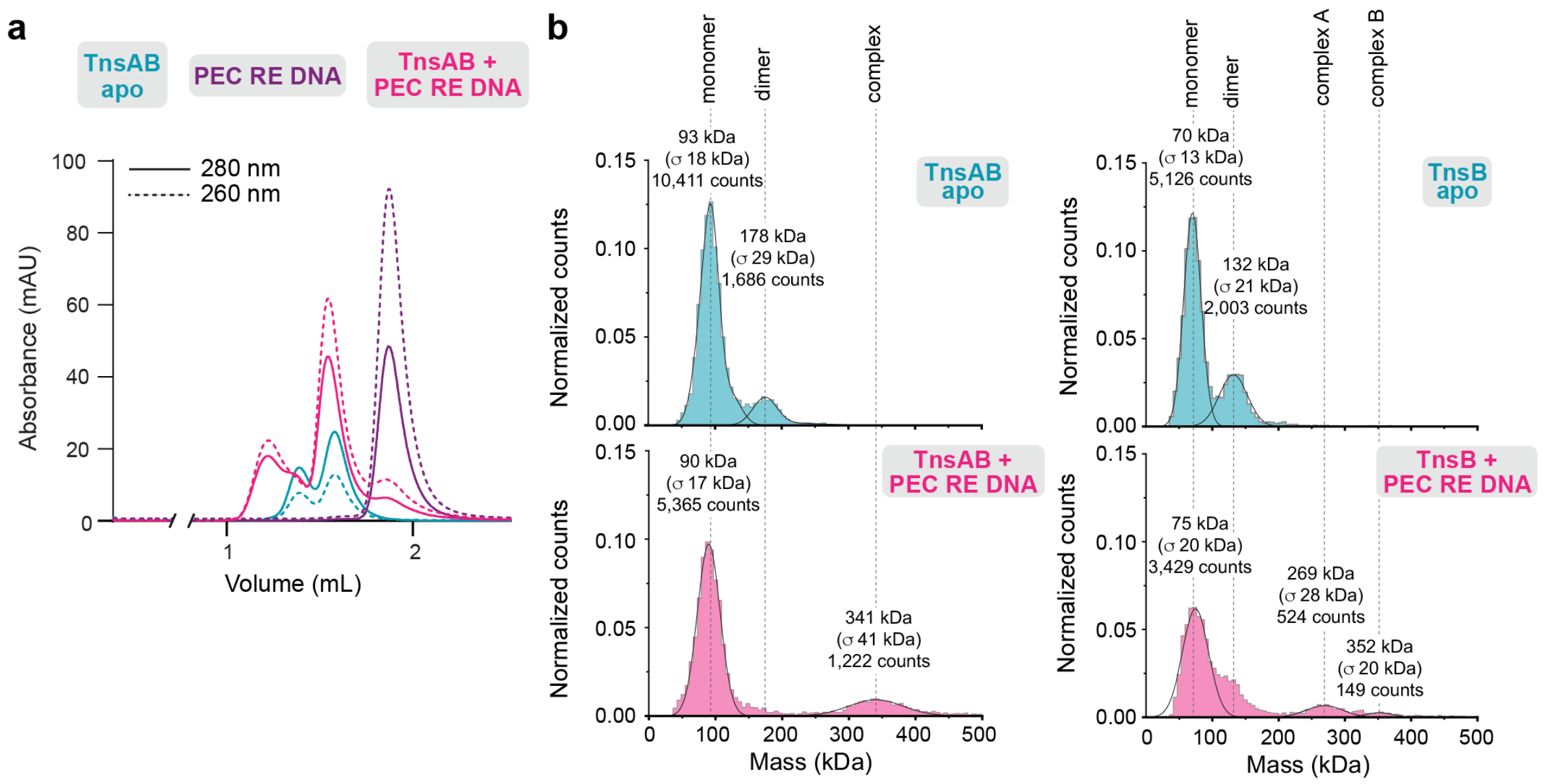

Supplementary Fig. 3 | Assembly of the *Pse*TnsAB PEC on right end DNA

(**a**) Size exclusion chromatography (SEC) profile of *Pse*TnsAB paired-end complex (PEC) reconstituted on transposon right end DNA (PEC RE DNA), used for structural analysis. (**b**) Mass photometry analysis of *Pse*TnsAB (left) and *Pse*TnsB (right) in its apo (top) and DNA-bound (bottom) states, with masses and respective particle population counts indicated. The theoretical molecular weight of a TnsAB monomer is 97.65 kDa, and of TnsB is 71.29 kDa.

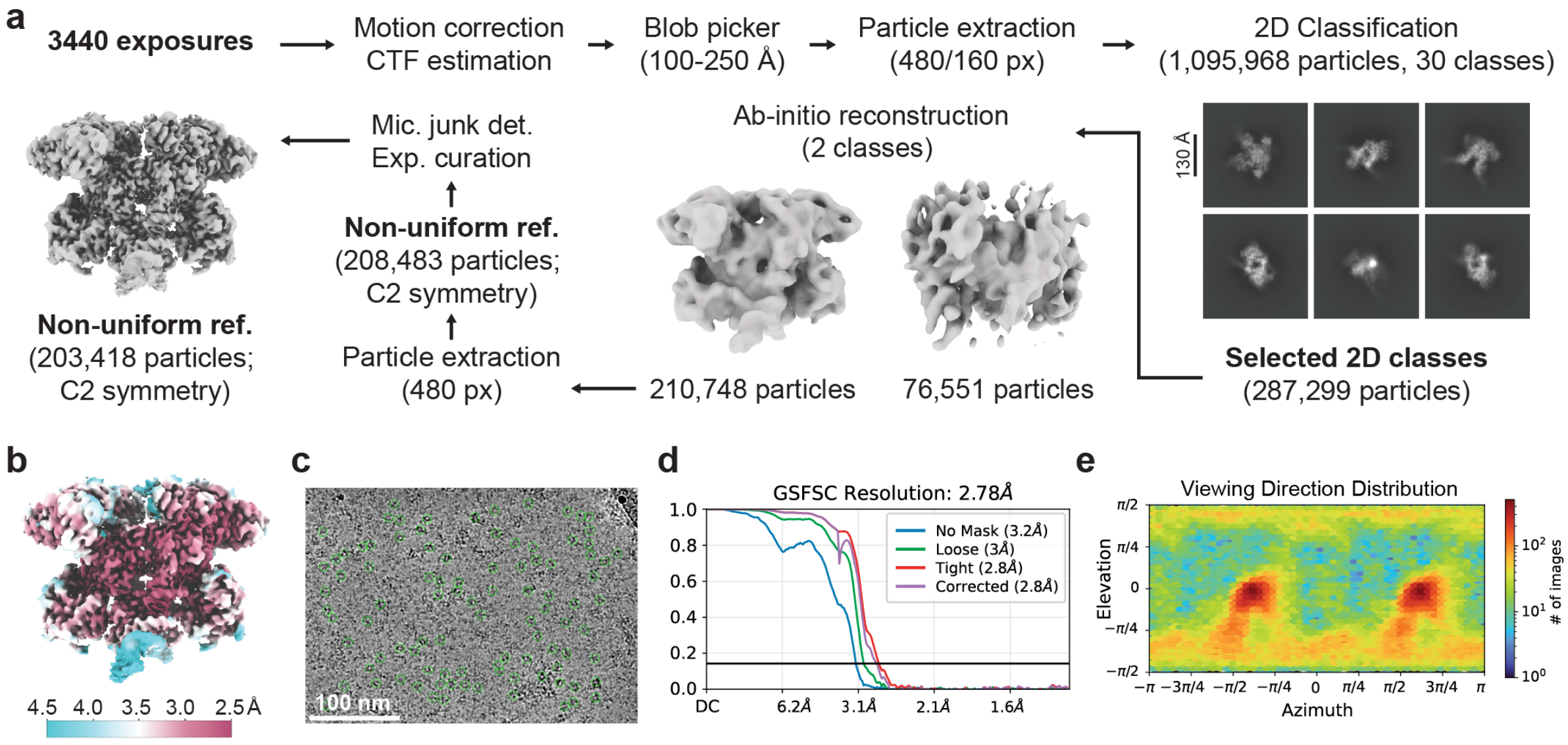

Supplementary Fig. 4 | Cryo-EM data processing workflow for the *Pse*TnsAB RE PEC in the presence of Mg^{2+}

(**a**) Cryo-EM image processing workflow for the *Pse*TnsAB paired-end complex (PEC) reconstituted on transposon right end (RE) DNA. (**b**) Cryo-EM density maps colored by local resolution. (**c**) Representative cryo-EM micrograph. Scale bar, 100 nm. (**d**) Fourier Shell Correlation (FSC) of the reconstruction calculated from two independently refined half-maps. The gold-standard cutoff (FSC = 0.143) is marked with a horizontal black line. (**e**) Angular distribution showing the range of observed particle orientations.

a

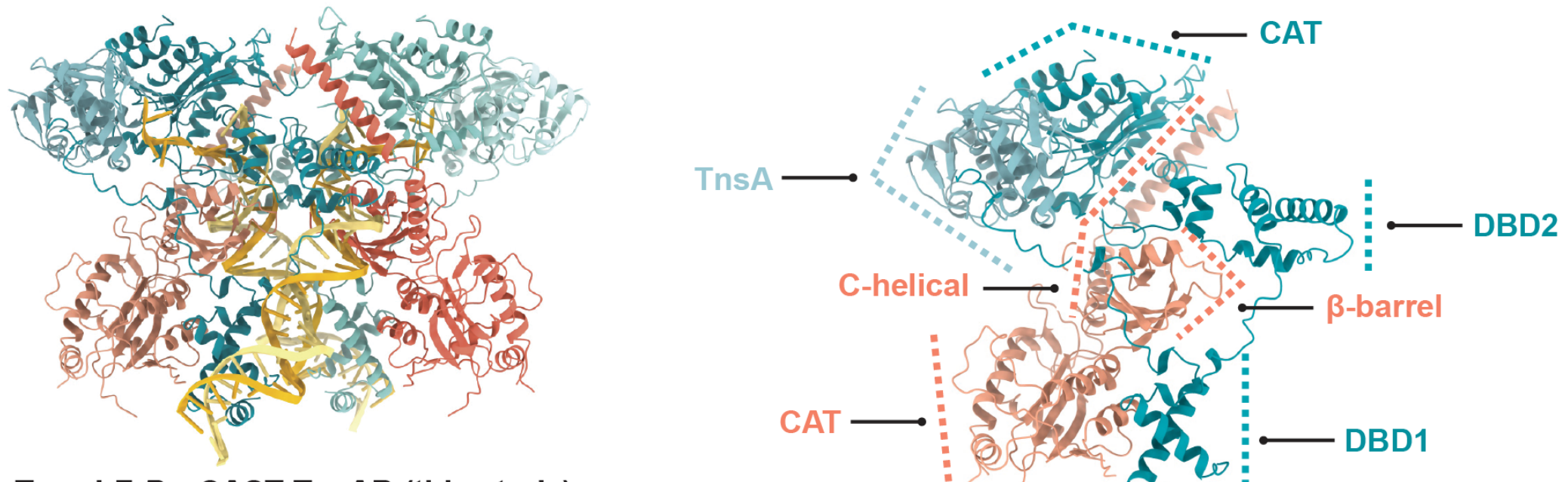

Type I-F *Pse*CAST TnsAB (this study)
Paired-End Complex
Transposon right end, $MgCl_2$

b

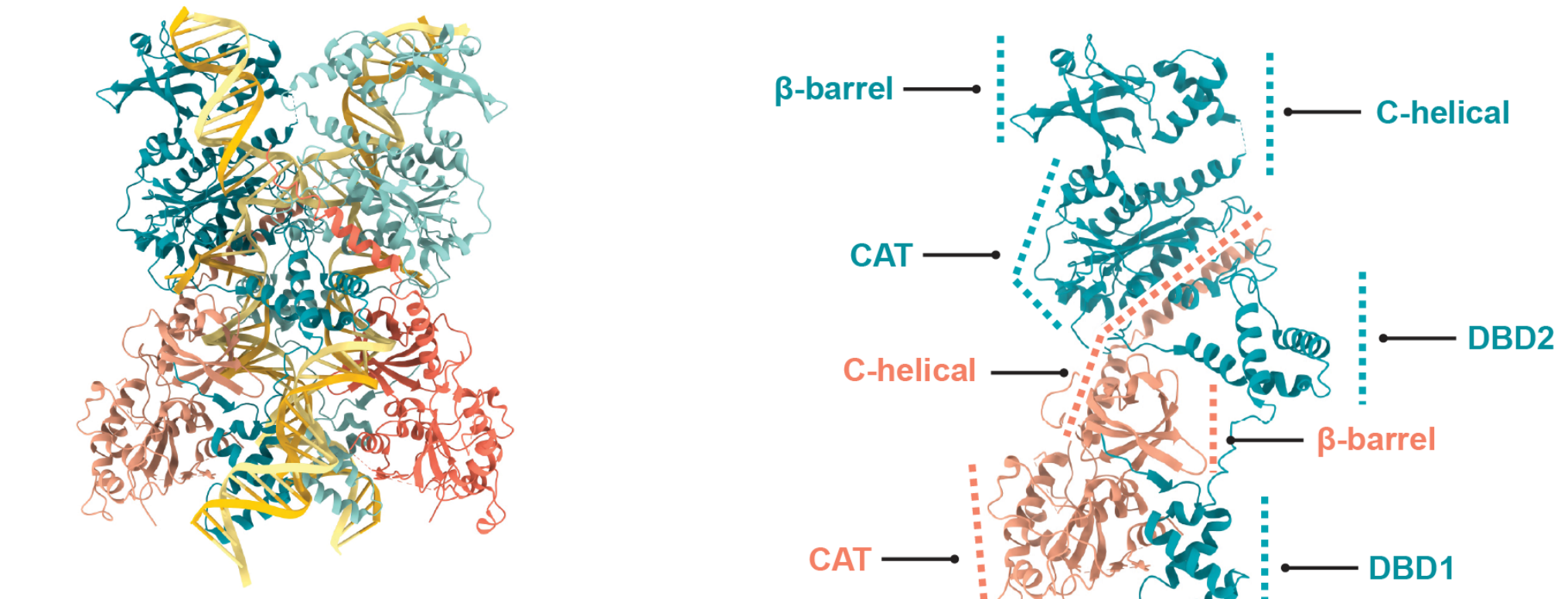

Type V-K *Sh*CAST TnsB (PDB 8EA3)
Strand-Transfer Complex
Transposon right and left ends, $MgCl_2$

c

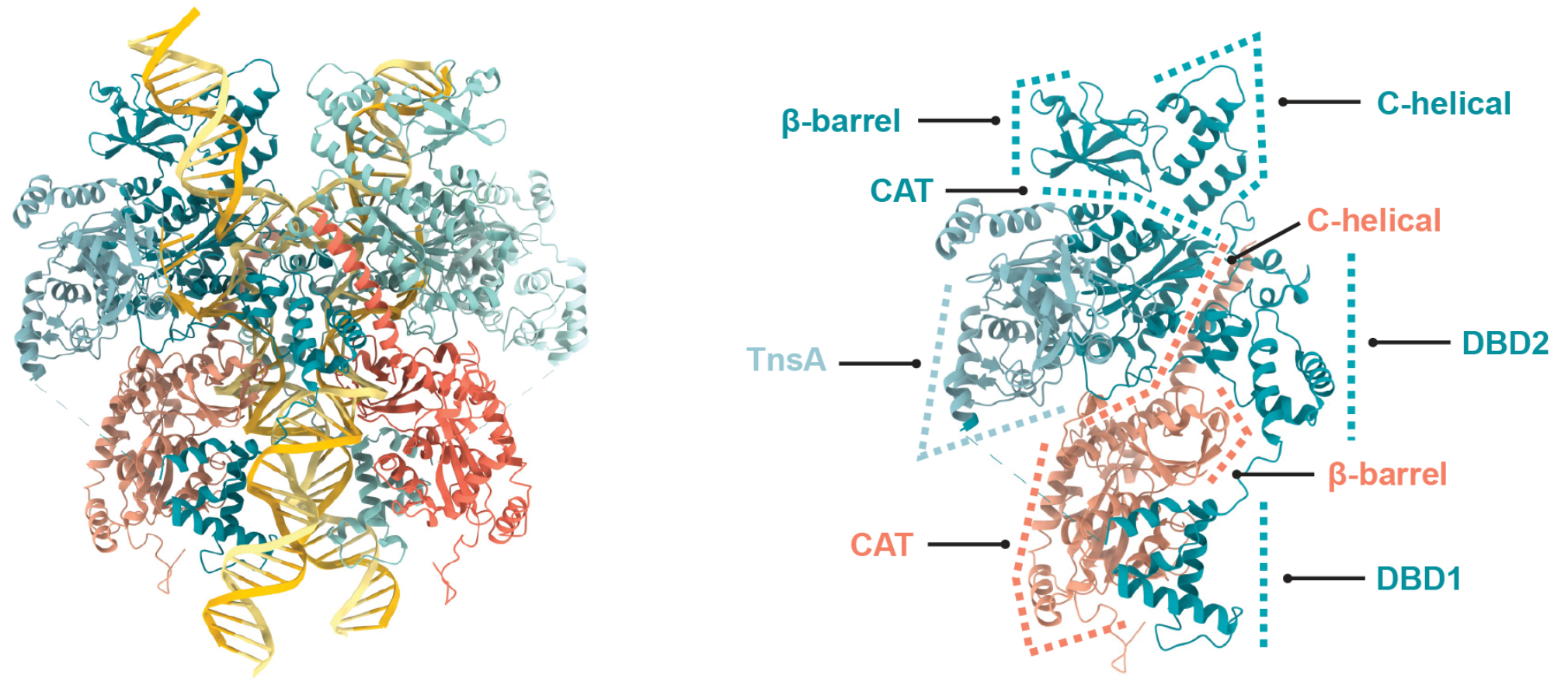

Type I-B2 *Pmc*CAST TnsAB (PDB 9BW1)
Strand-Transfer Complex
Transposon right and left ends, $MgCl_2$

d

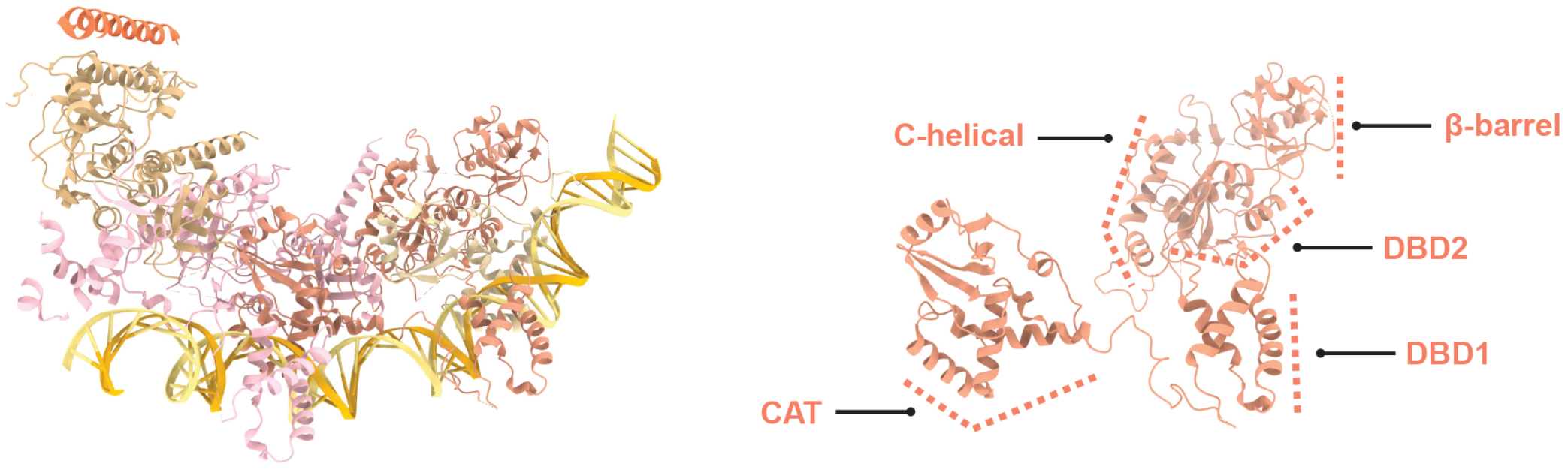

*Ec*Tn7 TnsB (PDB 7PIK)
Transposon-End Complex
Transposon right end

Supplementary Fig. 5 | Structural comparison of *Pse*TnsAB PEC, type V-K *Sh*TnsB STC, type I-B2 *Pmc*TnsAB STC, and Tn7 TnsB transposon-end complex

(**a**-**d**) Comparison of the *Pse*TnsAB paired-end complex (PEC) (**a**) with type V-K *Sh*TnsB[1] (**b**) and type I-B2 *Pmc*TnsAB[2] (**c**) strand-transfer complexes (STCs), and the *E. coli* Tn7 (*Ec*Tn7) TnsB transposon-end complex[3] (**d**). Left panels show overall architecture, right panels highlight domain differences. DBD, DNA binding domain; CAT, catalytic domain.

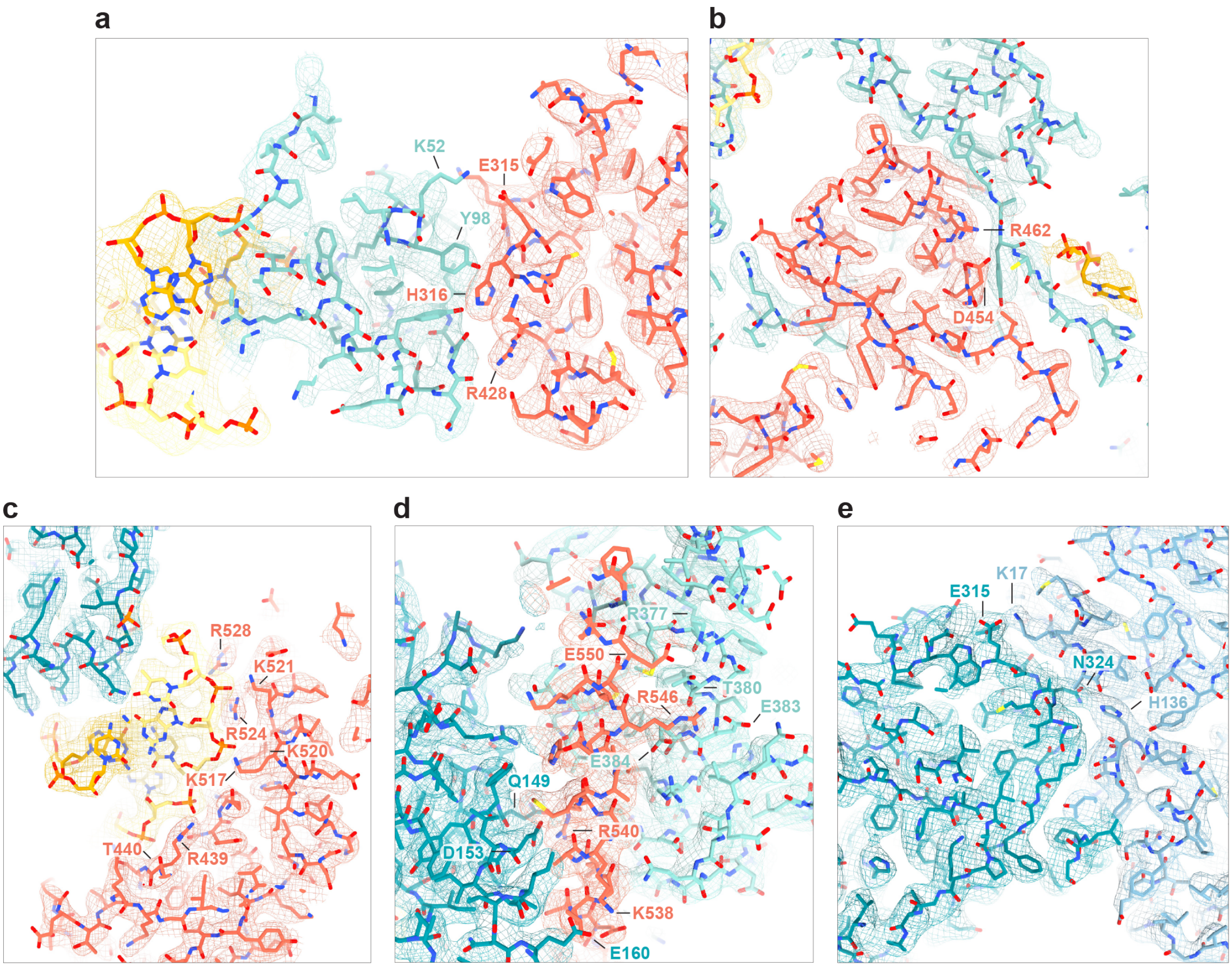

Supplementary Fig. 6 | Local model-to-map fit for regions involved in TnsB interprotomer interactions and TnsA-TnsB interfaces

Panels show zoom-in views of the structural regions corresponding to the indicated panels in **Fig. 3**, with the atomic model shown in stick representation overlaid with cryo-EM density displayed as a transparent mesh at the following contour levels: (**a**) 0.04, (**b**) 0.07, (**c**) 0.07, (**d**) 0.06, (**e**) 0.05. Residues highlighted in the corresponding panels of **Fig. 3** are labeled for reference, with consistent coloring. Panel correspondence: (**a**) **Fig. 3a**; (**b**) **Fig. 3b**; (**c**) **Fig. 3c**; (**d**) **Fig. 3d**; (**e**) **Fig. 3e**.

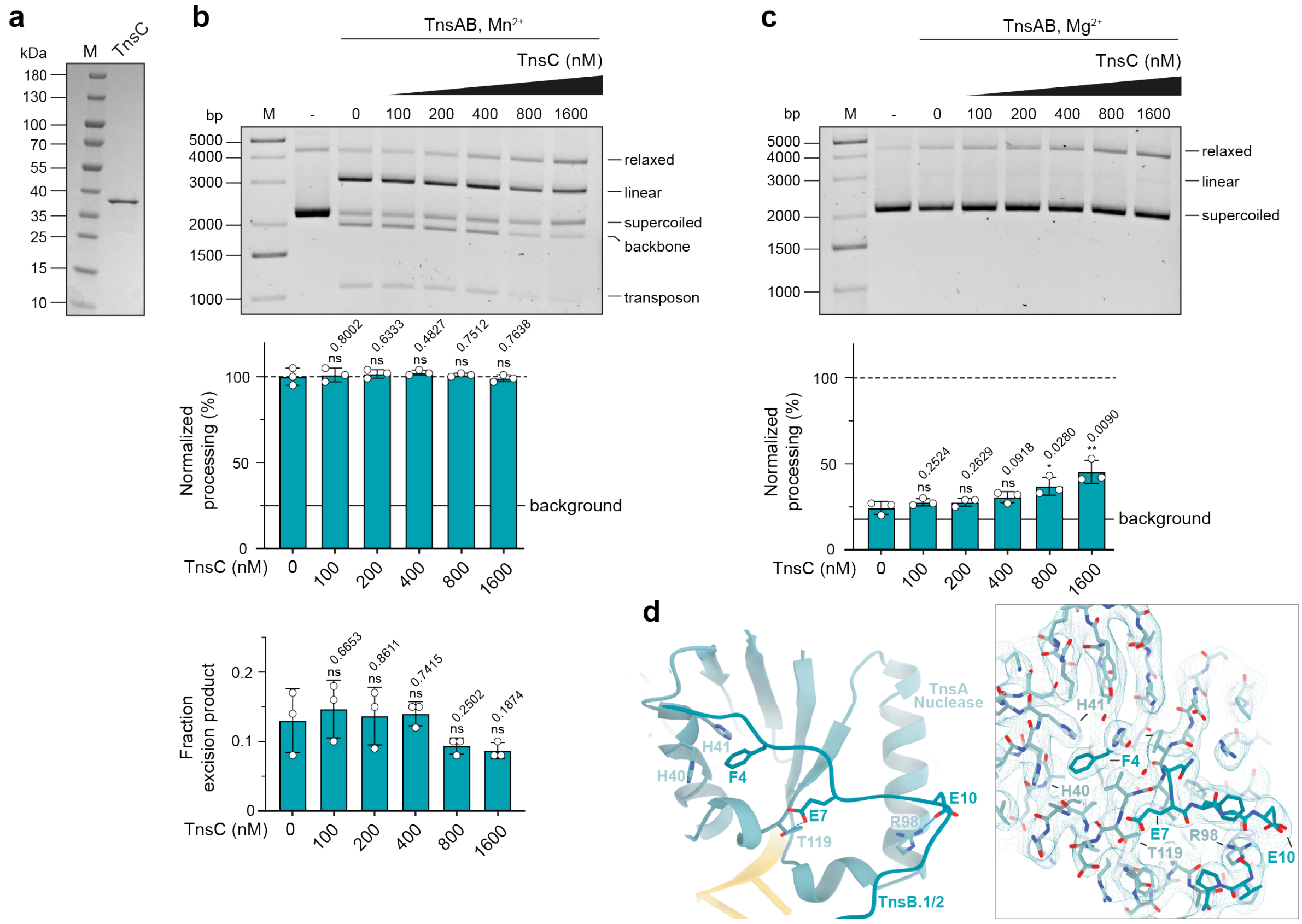

Supplementary Fig. 7 | *In vitro* cleavage activity in the presence of *Pse*TnsC and detailed view of TnsA-TnsB interactions

(**a**) SDS-PAGE analysis of purified *Pse*TnsC protein. M, marker. (**b-c**) *In vitro* excision assay with *Pse*TnsAB in the presence of increasing concentrations of *Pse*TnsC and either Mn^{2+} (**b**) or Mg^{2+} (**c**). Top: representative agarose gels showing resolved DNA species. -, no-protein control; M, marker. (**b**) Middle and bottom: Quantification of *in vitro* excision assay from three independent experiments (see **Methods** for details). Relative amounts of DNA species were determined by densitometry. Middle: Quantification of total DNA processing normalized to WT TnsAB in the presence of Mn^{2+} without TnsC. Data show mean ± s.d. (n = 3 independent experiments) of all processed DNA species. The solid line indicates the mean background level of processed plasmid in the no-protein control. Bottom: Quantification of excision. Data show mean ± s.d. (n = 3 independent experiments) of plasmid backbone (~2 kb-long excision product). (**c**) Bottom: Quantification from three independent experiments, as in (**b**, middle panel), for Mg^{2+} conditions. Total DNA processing is normalized to WT TnsAB in the presence of Mg^{2+} without TnsC. Statistical analysis was conducted using unpaired two-tailed t-test. P values above each graph indicate comparisons with the corresponding negative control. Significance is denoted as *P < 0.05; **P < 0.01; ns, not significant. Exact P values are shown above the corresponding bars. Source data are provided as a Source Data file. (**d**) Left: Close-up view of the specific contacts between the *Pse*TnsB N-terminus and *Pse*TnsA. Dashed lines represent interaction distances ≤ 3.5 Å. Right: Corresponding atomic model shown in stick representation overlaid with cryo-EM density shown as a transparent mesh and at a contour level of 0.004.

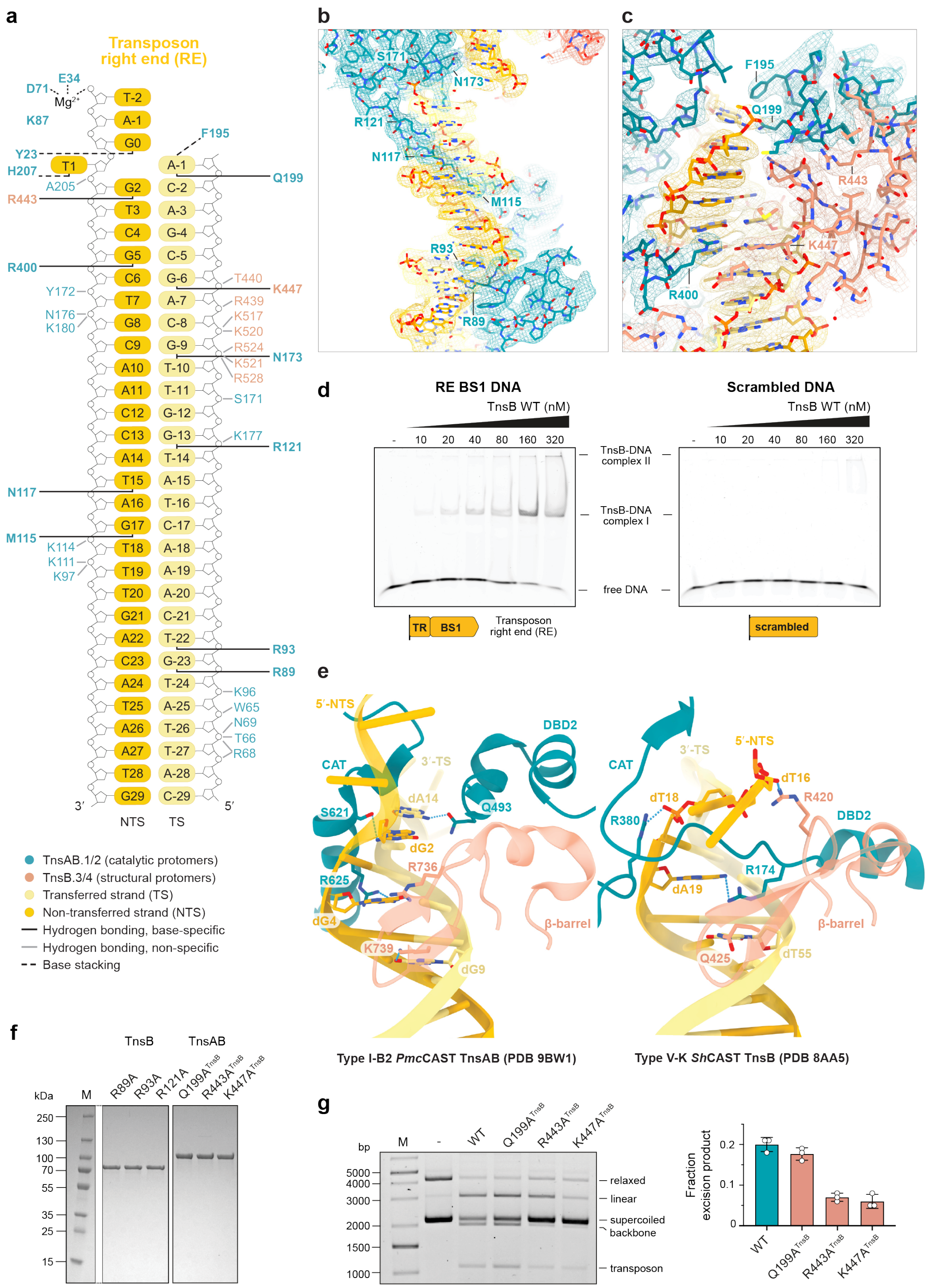
a
Transposon right end (RE)
NTS
TS
TnsAB.1/2 (catalytic protomers)
TnsB.3/4 (structural protomers)
Transferred strand (TS)
Non-transferred strand (NTS)
Hydrogen bonding, base-specific
Hydrogen bonding, non-specific
Base stacking
b
c
d
RE BS1 DNA
Scrambled DNA
TnsB WT (nM)
TnsB-DNA complex II
TnsB-DNA complex I
free DNA
Transposon right end (RE)
scrambled
e
Type I-B2 PmcCAST TnsAB (PDB 9BW1)
Type V-K ShCAST TnsB (PDB 8AA5)
f
TnsB
TnsAB
kDa
g
relaxed
linear
supercoiled backbone
transposon
Fraction excision product

Supplementary Fig. 8 | Transposon right end DNA binding by *Pse*TnsAB

(**a**) Schematic representation of interactions between *Pse*TnsAB and transposon right end DNA within the paired-end complex. (**b-c**) Panels show a zoom-in views of the structural regions corresponding to the indicated panels in **Fig. 4**, with the atomic model shown in stick representation overlaid with cryo-EM density displayed as a transparent mesh at a contour level of 0.05 for (**b**) and 0.06 for (**c**). Residues highlighted in the corresponding panels of **Fig. 4** are labeled for reference, with consistent coloring. Panel correspondence: (**b**) **Fig. 4a**; (**c**) **Fig. 4d**. (**d**) Analysis of *Pse*TnsB-DNA interactions by electrophoretic mobility shift assays (EMSA). Each panel shows a representative EMSA for indicated DNA. Left, RE BS1 DNA comprising the native BS1 (TnsB-binding site 1) and terminal repeat (TR) sequences of the transposon right end (RE); right, scrambled DNA sequence. Cy5-labeled DNAs were incubated with increasing concentrations of TnsB. Protein-DNA complexes are marked on the side of each native PAGE gel. -, no-protein control; WT, wild-type. (**e**) Structural comparison of TR recognition in the type I-B2 *Pmc*TnsAB[2] and the type V-K *Sh*TnsB[4] strand-transfer complexes. DBD, DNA binding domain; CAT, catalytic domain. TS, transferred strand; NTS, non-transferred strand. Dashed lines represent interaction contacts with interatomic distances ≤ 3.5 Å. (**f**) SDS-PAGE of purified mutant *Pse*TnsB and *Pse*TnsAB proteins with substitutions in the DNA binding interface of TnsB. (**g**) *In vitro* excision assay with *Pse*TnsAB variants carrying mutations in TR binding residues of TnsB. Left: Representative agarose gel of resolved DNA species; -, no-protein control. Right: quantification of excision from three independent experiments. Relative amounts of plasmid backbone (~2 kb-long excision product) were determined by densitometry. Data represent mean ± s.d. (n = 3 independent experiments). See **Methods** for details. M, marker. Source data are provided as a Source Data file.

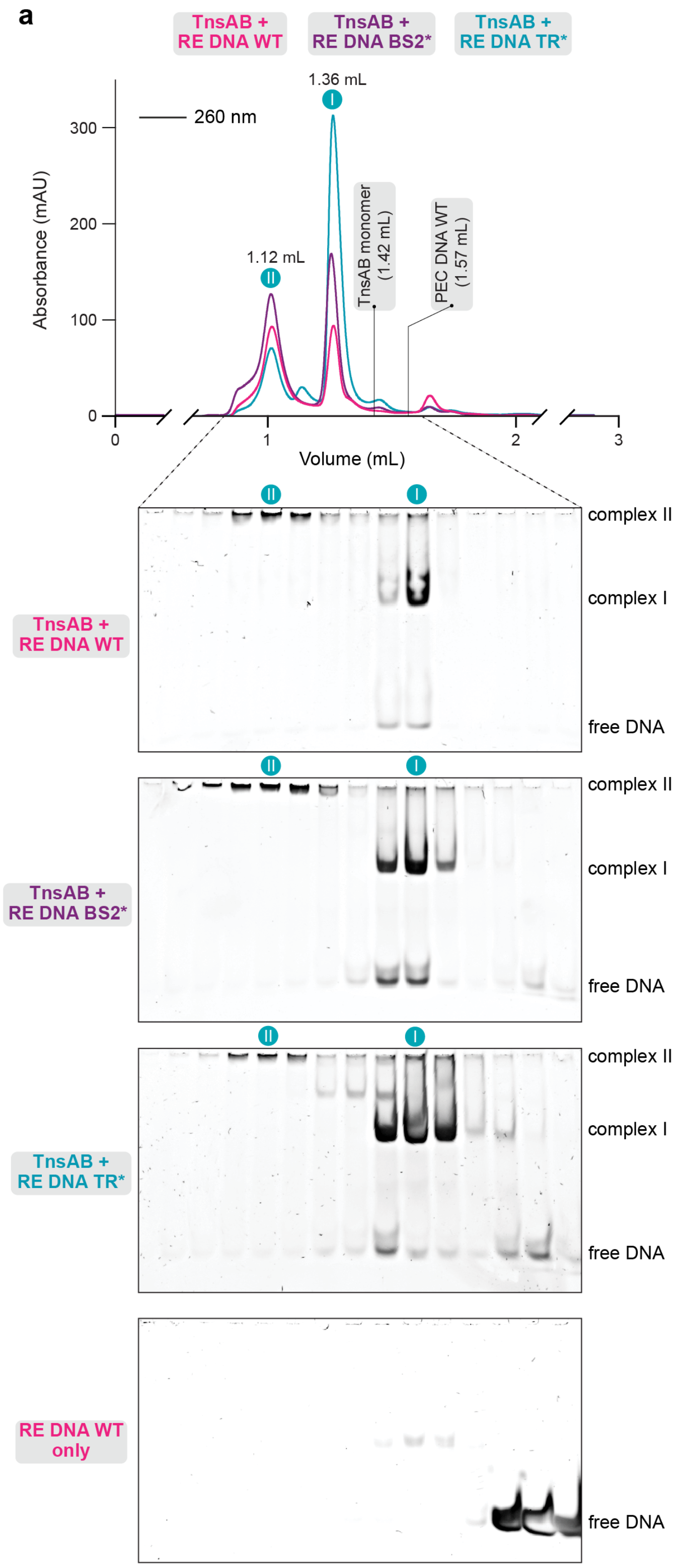

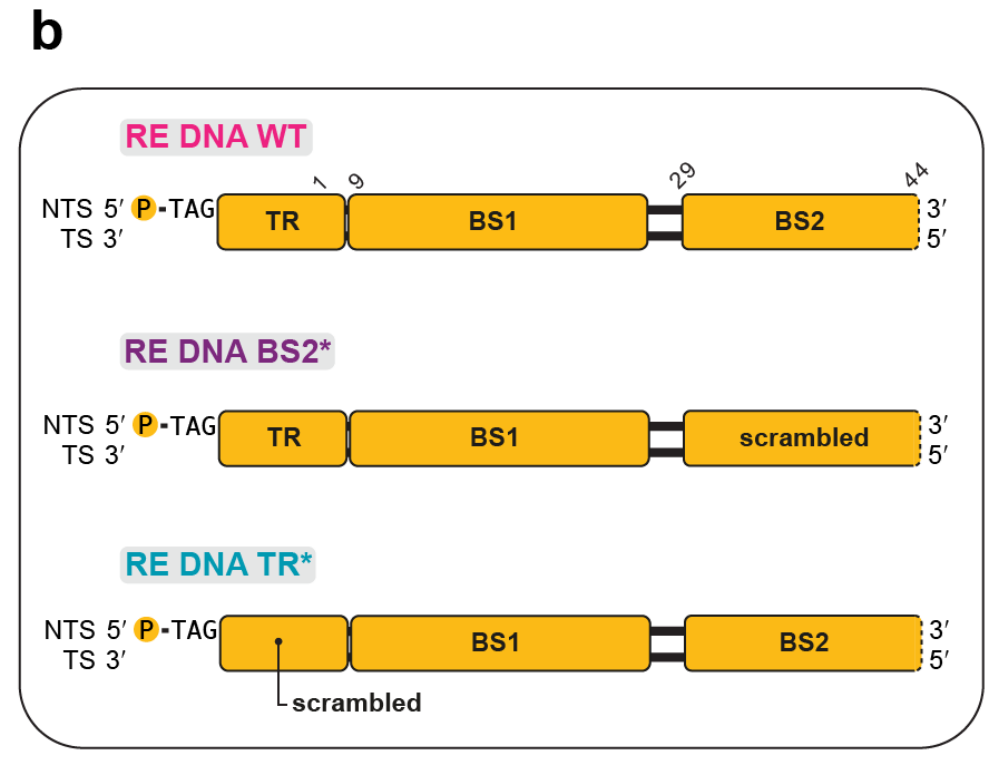

c

TnsAB + RE DNA	mAU of peak II	mAU of peak I	Ratio mAU II / mAU I
WT	93.1	94.2	0.96
BS2*	127.0	169.1	0.75
TR*	70.9	312.9	0.23

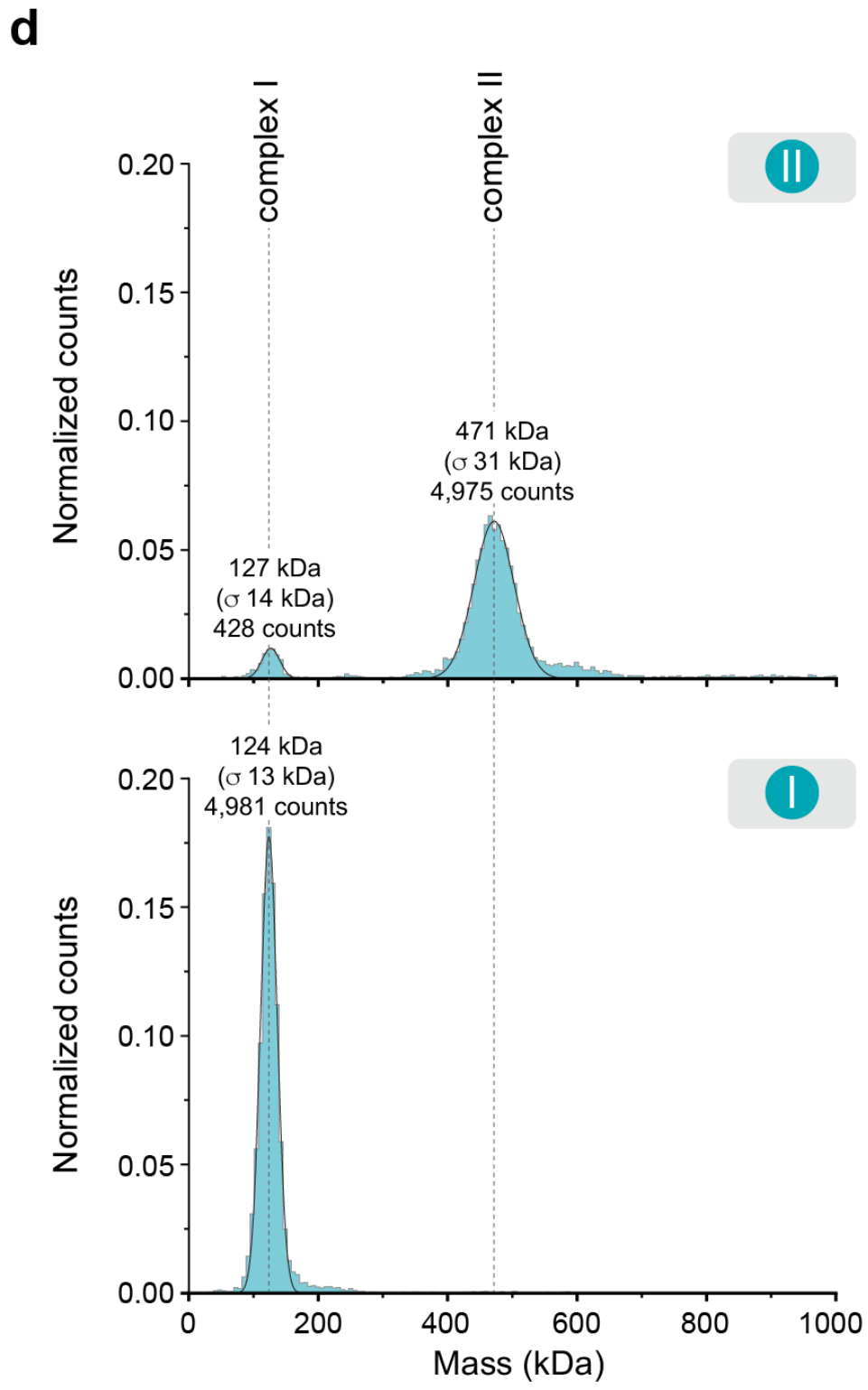

Supplementary Fig. 9 | DNA sequence requirements for *Pse*TnsAB complex assembly

(**a**) Size-exclusion chromatography (SEC) profiles of reconstituted *Pse*TnsAB-DNA complexes and analysis of elution fractions by native PAGE. Elution volumes for protein-only and DNA-only controls are indicated on the chromatogram for reference. (**b**) Schematic of the right end (RE) DNAs used, indicating the TnsB binding sites (BS1 and BS2) and the terminal repeat (TR) sequences of the transposon right end (RE). Scrambled DNA sequences are highlighted. NTS, non-transferred strand;

TS, transferred strand. (**c**) Table summarizing SEC analysis of complex I and complex II formation, based on absorbance at 260 nm. (**d**) Mass photometry analysis of PseTnsAB complex II (top) and complex I (bottom), with measured masses and corresponding particle population distributions indicated. The theoretical molecular weight of a TnsAB monomer bound to a single RE DNA is 125.8 kDa, and that of a TnsAB tetramer bound to two RE DNA substrates is 447.0 kDa. Source data are provided as a Source Data file.

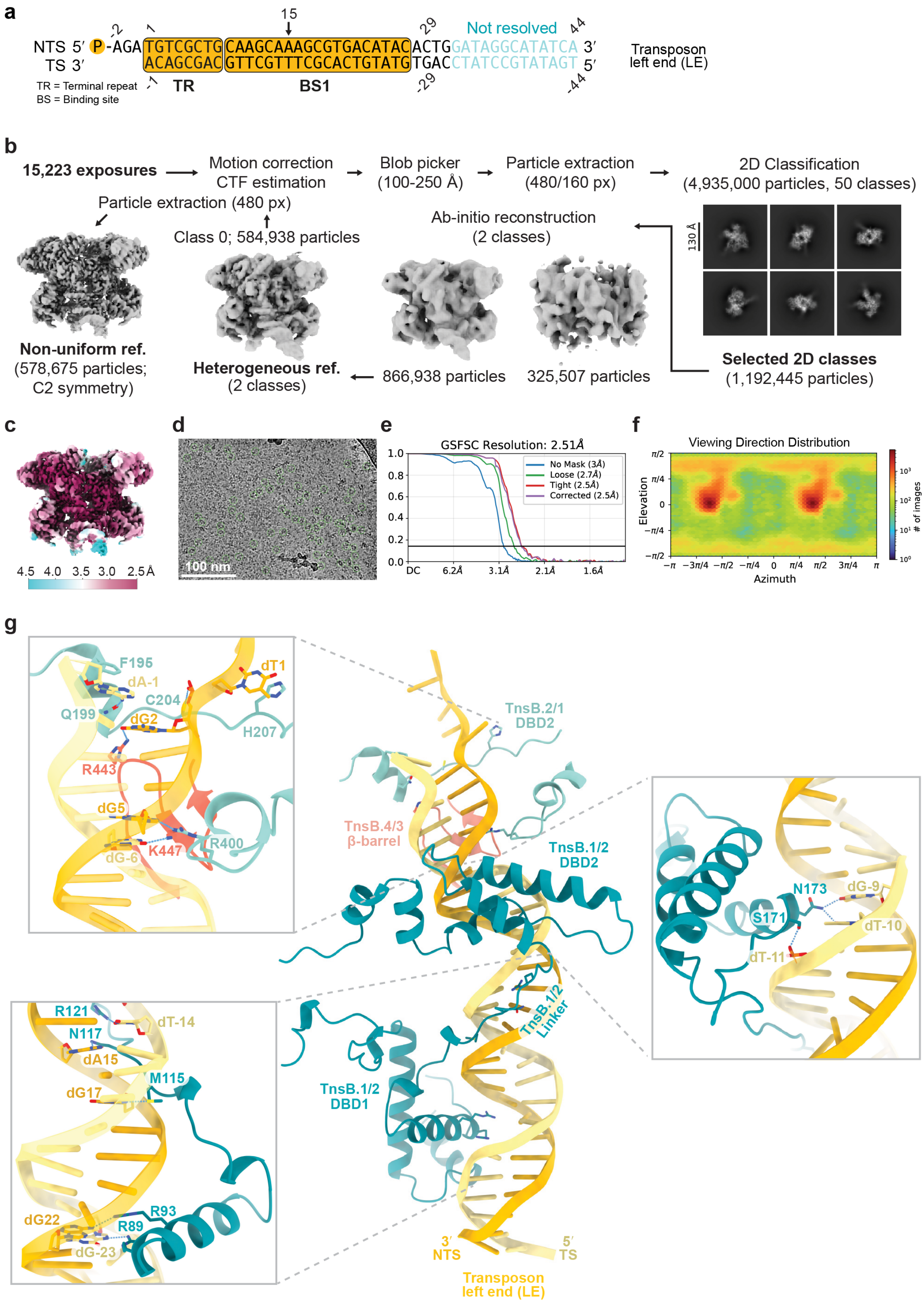

a
NTS 5′ P-AGA TGTCGCTG CAAGCAAAGCGTGACATAC ACTG GATAGGCATATCA 3′
TS 3′ ACAGCGAC GTTCGTTTCGCACTGTATG TGAC CTATCCGTATAGT 5′
-2
1
15
29
44
-1
-29
-44
Not resolved
TR
BS1
TR = Terminal repeat
BS = Binding site
Transposon
left end (LE)
b
15,223 exposures
Motion correction
CTF estimation
Blob picker
(100-250 Å)
Particle extraction
(480/160 px)
2D Classification
(4,935,000 particles, 50 classes)
Particle extraction (480 px)
Class 0; 584,938 particles
Ab-initio reconstruction
(2 classes)
130 Å
Non-uniform ref.
(578,675 particles;
C2 symmetry)
Heterogeneous ref.
(2 classes)
866,938 particles
325,507 particles
Selected 2D classes
(1,192,445 particles)
c
4.5 4.0 3.5 3.0 2.5Å
d
100 nm
e
GSFSC Resolution: 2.51Å
No Mask (3Å)
Loose (2.7Å)
Tight (2.5Å)
Corrected (2.5Å)
1.0
0.8
0.6
0.4
0.2
0.0
DC
6.2Å
3.1Å
2.1Å
1.6Å
f
Viewing Direction Distribution
Elevation
Azimuth
# of images
g
F195
dA-1
dT1
C204
Q199
dG2
H207
R443
dG5
R400
K447
dG-6
TnsB.2/1
DBD2
TnsB.4/3
β-barrel
TnsB.1/2
DBD2
N173
dG-9
S171
dT-10
dT-11
TnsB.1/2
Linker
R121
dT-14
N117
dA15
M115
dG17
TnsB.1/2
DBD1
dG22
R93
R89
dG-23
3′
NTS
5′
TS
Transposon
left end (LE)

Supplementary Fig. 10 | Cryo-EM analysis of the *Pse*TnsAB LE PEC in the presence of Mg^{2+}

(**a**) Schematic of the transposon left end (LE) DNA substrate used for paired-end complex (PEC) reconstitution, showing the TnsB-binding site BS1, and the terminal repeat (TR) sequences. NTS, non-transferred strand. TS, transferred strand. A15 is indicated with an arrow. (**b**) Cryo-EM image processing workflow. (**c**) Cryo-EM density maps colored by local resolution. (**d**) Representative cryo-EM micrograph. Scale bar, 100 nm. (**e**) Fourier Shell Correlation (FSC) of the reconstruction calculated from two independently refined half-maps. The gold-standard cutoff (FSC = 0.143) is marked with a horizontal black line. (**f**) Angular distribution showing the range of observed particle orientations. (**g**) Detailed views of sequence-specific protein-DNA interactions mediated by the DBD2 (top), linker (middle) and DBD1 (bottom) of the TnsB catalytic protomers on the PEC LE DNA. Dashed lines represent interaction distances ≤ 3.5 Å.

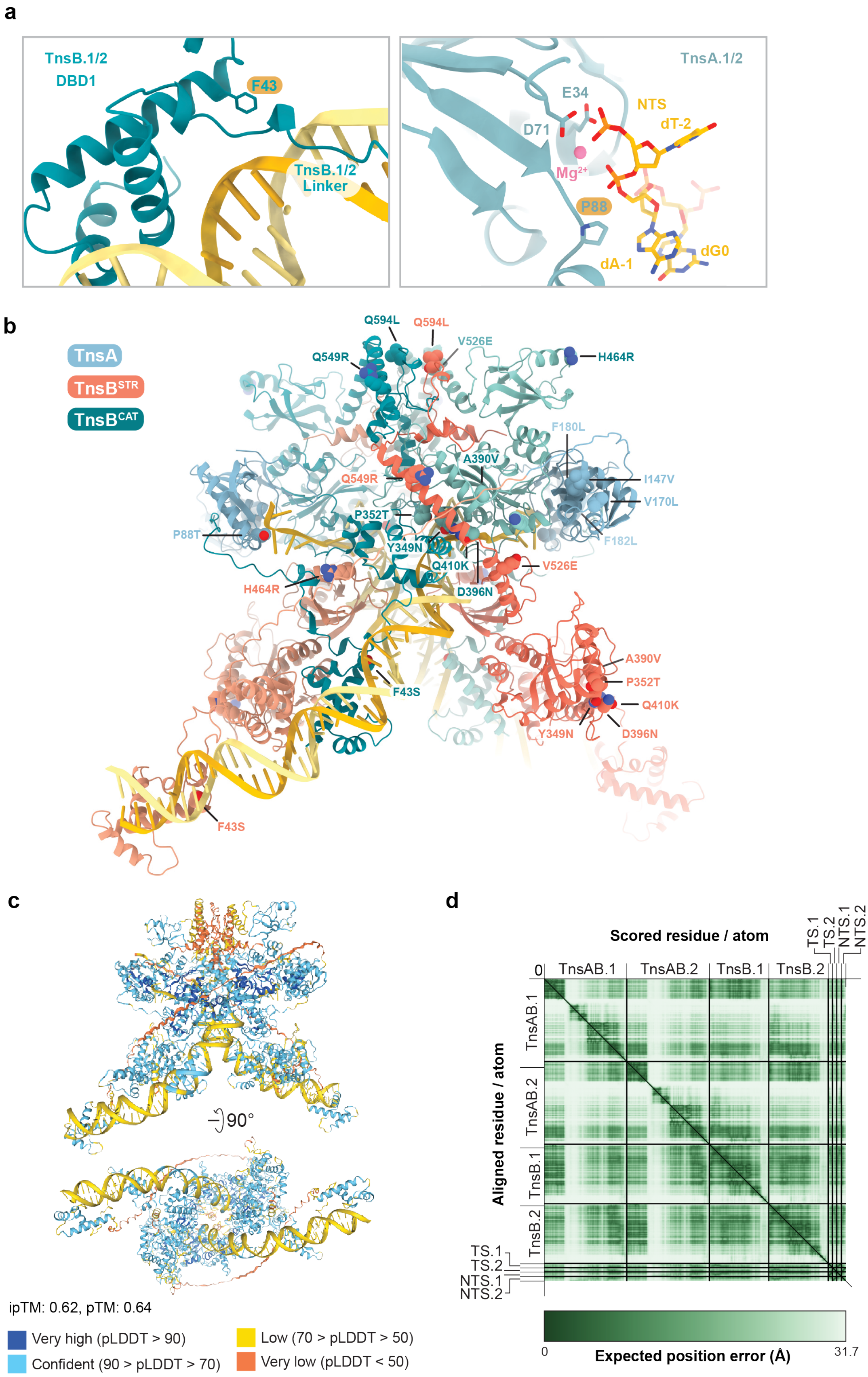
a
TnsB.1/2
DBD1
F43
TnsB.1/2
Linker
TnsA.1/2
E34
NTS
D71
dT-2
Mg2+
P88
dA-1
dG0
b
TnsA
TnsBSTR
TnsBCAT
Q594L
Q594L
V526E
H464R
Q549R
F180L
A390V
Q549R
I147V
V170L
P352T
P88T
F182L
Y349N
Q410K
V526E
H464R
D396N
A390V
P352T
F43S
Q410K
Y349N
D396N
F43S
c
90°
ipTM: 0.62, pTM: 0.64
Very high (pLDDT > 90)
Low (70 > pLDDT > 50)
Confident (90 > pLDDT > 70)
Very low (pLDDT < 50)
d
Scored residue / atom
TS.1
TS.2
NTS.1
NTS.2
0
TnsAB.1
TnsAB.2
TnsB.1
TnsB.2
Aligned residue / atom
TnsAB.1
TnsAB.2
TnsB.1
TnsB.2
TS.1
TS.2
NTS.1
NTS.2
0
Expected position error (Å)
31.7

Supplementary Fig. 11 | *In silico* mapping of amino acid substitutions in evolved *Pse*TnsAB

(**a**) Detailed view of selected residues mutated in evolved (evo)TnsAB mapped onto the *Pse*TnsAB paired-end complex (PEC) structure. The atomic model corresponds to the PEC assembled on right end DNA in the presence of Mg^{2+}. Phe43^{TnsB} and Pro88^{TnsA} are highlighted in orange. NTS, non-transferred strand. (**b**) AlphaFold 3[5] model of evoTnsAB PEC assembled on the same right end DNA substrate used for experimental structural determination. TnsA, structural (TnsB^{STR}) and catalytic TnsB (TnsB^{CAT}) protomers are highlighted in distinct colours (see legend). Residues mutated in evoTnsAB relative to TnsAB WT are shown as spheres. (**c**) Predicted model shown in two orthogonal orientations, coloured by per-residue confidence (pLDDT). Interface and overall model confidence: ipTM = 0.62, pTM = 0.64. Model is provided as **Supplementary Data 4**. (**d**) Predicted aligned error (PAE) plot for the model. Source data are provided as a Source Data file.

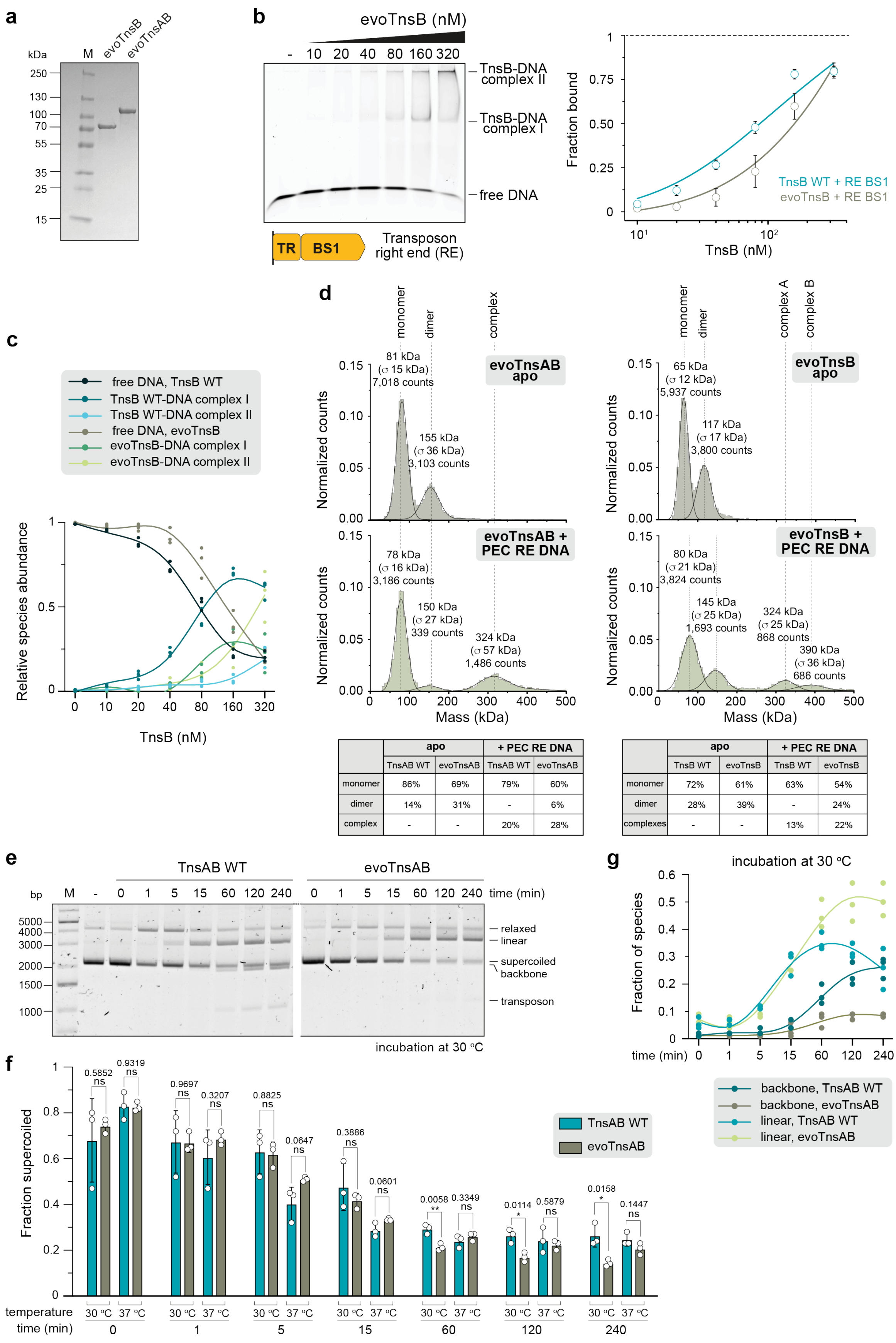

	apo		+ PEC RE DNA	
	TnsAB WT	evoTnsAB	TnsAB WT	evoTnsAB
monomer	86%	69%	79%	60%
dimer	14%	31%	-	6%
complex	-	-	20%	28%

	apo		+ PEC RE DNA	
	TnsB WT	evoTnsB	TnsB WT	evoTnsB
monomer	72%	61%	63%	54%
dimer	28%	39%	-	24%
complexes	-	-	13%	22%

Supplementary Fig. 12 | *In vitro* analysis of evolved *Pse*TnsAB

(**a**) SDS-PAGE analysis of purified evoTnsAB and evoTnsB proteins. M, marker. (**b**) EMSA analysis of evoTnsB-DNA interactions compared with wild-type (WT). The Cy5-labeled substrate was RE BS1 DNA containing the native BS1 (TnsB-binding site 1) and terminal repeat (TR) sequences of the transposon right end (RE), as in **Supplementary Fig. 8d**. Left: representative native PAGE gel for evoTnsB, with protein-DNA complexes indicated. -, no-protein control. Right: densitometric quantification showing the fraction of bound DNA versus protein concentration (mean ± s.d., *n* = 3 independent experiments). (**c**) Quantification of relative species abundances from three independent EMSA experiments. (**d**) Mass photometry of evoTnsAB and evoTnsB in apo and DNA-bound states using transposon right end DNA (PEC RE DNA), with measured masses and particle population counts. A comparison with parameters for WT protein is provided in the accompanying table. (**e-g**) *In vitro* time-course excision assays of WT and evo*Pse*TnsAB variants at different temperatures. (**e**) Representative agarose gel showing resolved DNA species for assays performed at 30 °C. (**f-g**) Densitometric quantification of supercoiled plasmid (mean ± s.d.) (**f**), plasmid backbone (~2 kb-long excision product) and linearized plasmid (~3 kb-long single-end cleaved product) (**g**) (*n* = 3 independent experiments). Statistical analysis was conducted using unpaired two-tailed *t*-tests. Significance is denoted as *P < 0.05; **P < 0.01; ns, not significant. Exact P values are shown above the corresponding bars (see **Methods** for details). Source data are provided as a Source Data file.

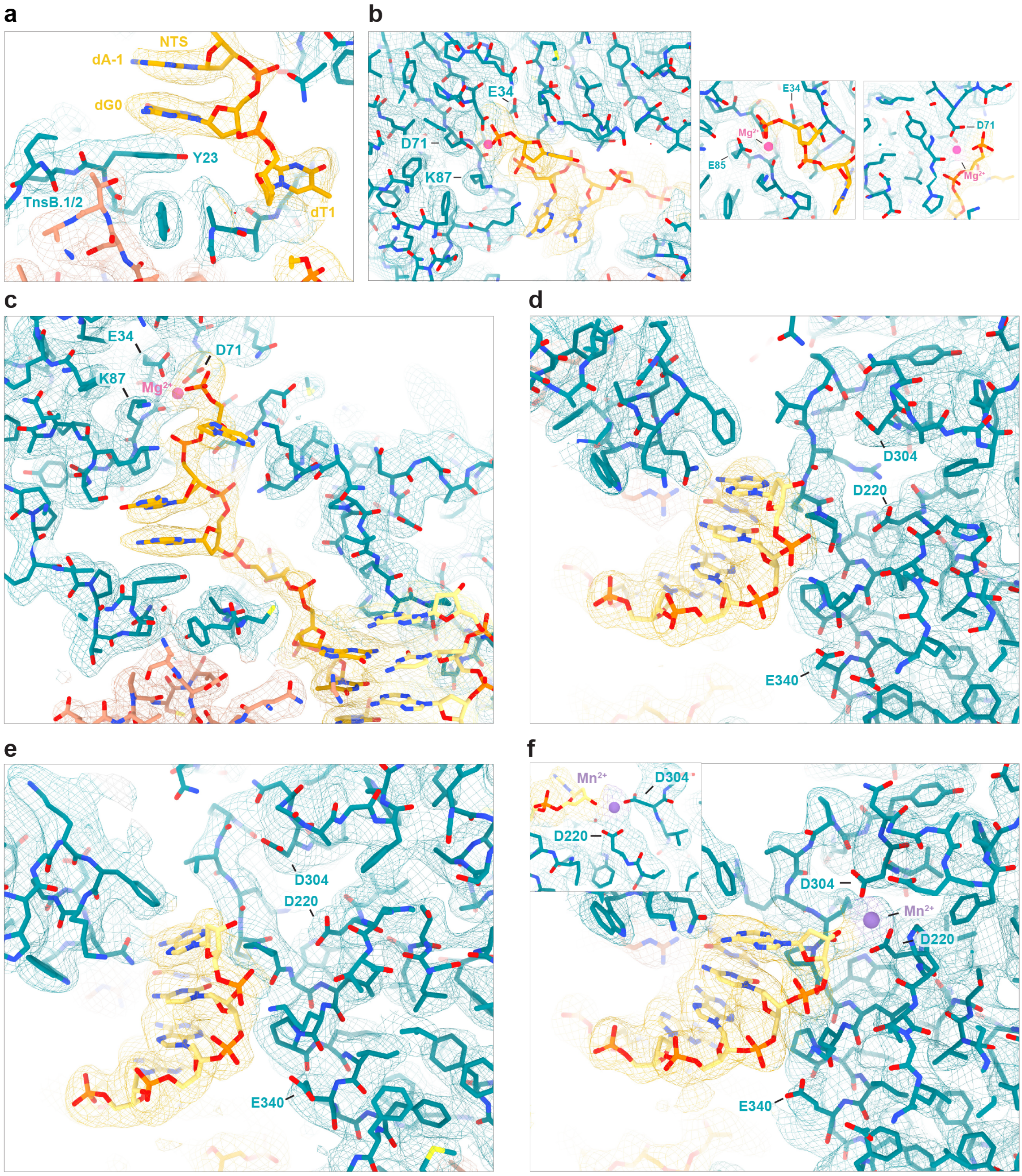

a
NTS
dA-1
dG0
Y23
TnsB.1/2
dT1
b
E34
D71
K87
E34
Mg2+
E85
D71
Mg2+
c
E34
D71
K87
Mg2+
d
D304
D220
E340
e
D304
D220
E340
f
Mn2+
D304
D220
D304
Mn2+
D220
E340

Supplementary Fig. 13 | Structural details of TnsA and TnsB active sites

(**a**) Zoom-in view of the TnsB N-terminal tail domain stabilizing the 3-nucleotide 5′ overhang of the NTS non-transferred strand (NTS), accommodated in the TnsA active site. The atomic model is shown in stick representation overlaid with cryo-EM density displayed as a transparent mesh at a contour level of 0.05. (**b-c**) Zoom-in views of the TnsA active site in PEC structures assembled on right end DNA (**b**, corresponding to **Fig. 5b**) and left end DNA (**c**) in the presence of Mg^{2+}. Atomic models are shown in stick representation overlaid with cryo-EM density displayed as a transparent mesh at contour levels of 0.04 (**b**, left panel and **c**) and 0.06 (**b**, right panel). (**d-e**) Zoom-in views of the TnsB active site in PEC structures assembled on right end DNA (**d**, corresponding to **Fig. 5c**) and left end DNA (**e**) in the presence of Mg^{2+}. Atomic models are shown in stick representation overlaid with cryo-EM density displayed as a transparent mesh at contour levels of 0.05 (**d**) and 0.04 (**e**). (**f**) Zoom-in view of the TnsB active site in the PEC structure assembled on right end DNA in the presence of Mn^{2+} (corresponding to **Fig. 5d**). The atomic model is shown in stick representation overlaid with cryo-EM density displayed as a transparent mesh at a contour level of 0.05. Residues highlighted in the corresponding panels of Fig. 5 are labeled for reference, with consistent coloring.

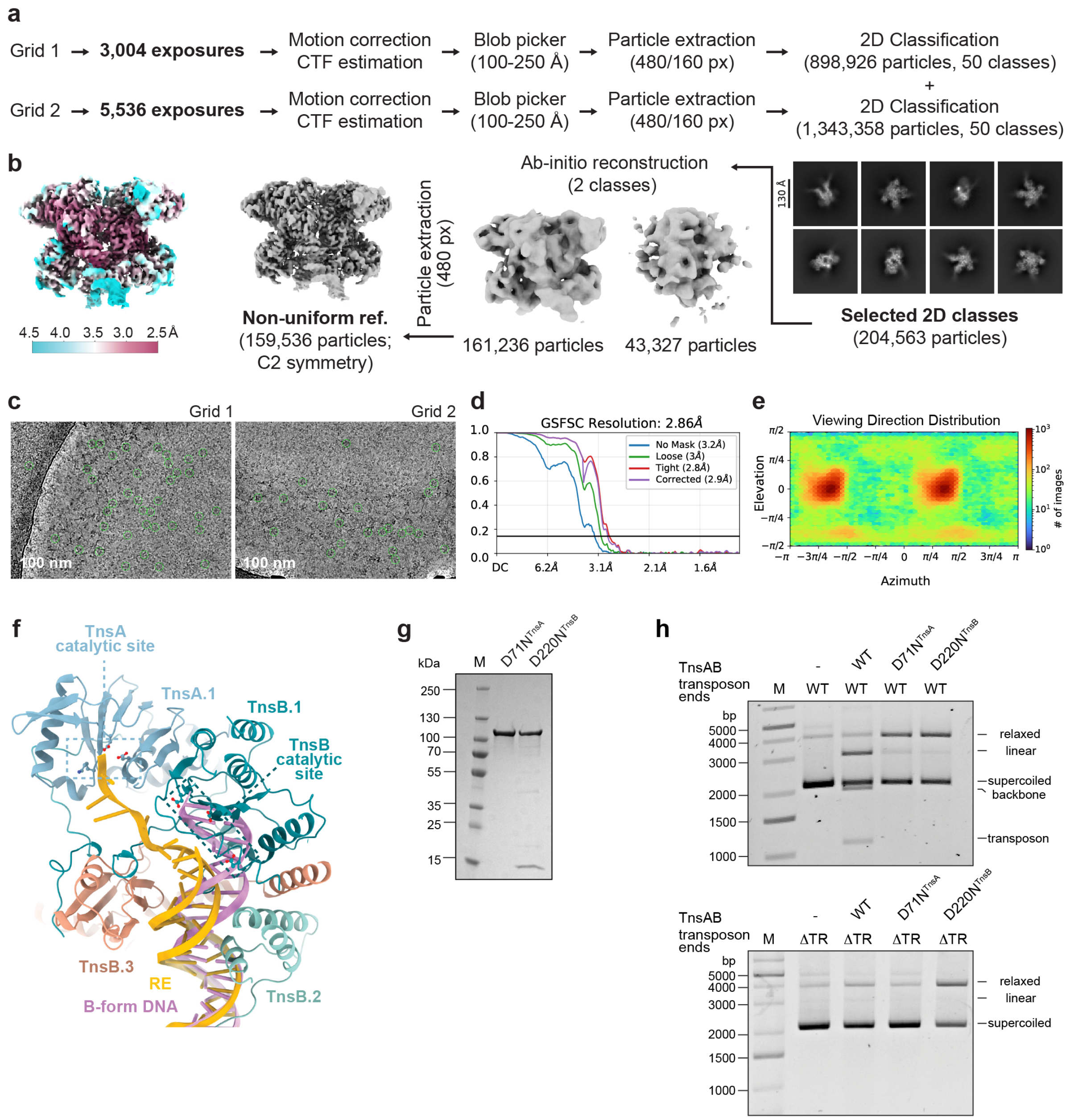

Supplementary Fig. 14 | Cryo-EM analysis of *Pse*TnsAB RE PEC in the presence of Mn^{2+}

(**a**) Cryo-EM image processing workflow for the *Pse*TnsAB paired-end complex (PEC) assembled on transposon right end (RE) DNA in the presence of Mn^{2+}. (**b**) Cryo-EM density maps colored by local resolution. (**c**) Representative cryo-EM micrographs. Scale bars, 100 nm. (**d**) Fourier Shell Correlation (FSC) of the reconstruction calculated from two independently refined half-maps. The gold-standard cutoff (FSC = 0.143) is marked with a horizontal black line. (**e**) Angular distribution of observed particle orientations. (**f**) Canonical B-form DNA cannot be accommodated in both TnsA and TnsB active sites without steric clashes. The atomic model corresponds to the PEC assembled on right end DNA in the presence of Mg^{2+}. (**g**) SDS-PAGE analysis of purified *Pse*TnsAB proteins with mutations in the TnsA or TnsB subunit. (**h**) *In vitro* excision assay with *Pse*TnsAB wild-type (WT) protein and catalytic site mutants. Representative agarose gels of reactions with plasmids containing either wild-type transposon ends (top) or lacking terminal repeat (TR) sequences on both LE and RE (bottom); -, no-protein control; M, marker. Source data are provided as a Source Data file.

Supplementary Tables

Supplementary Table 1 | Cryo-EM data collection, refinement and validation statistics

ID, EMDB and PDB codes	Cryo-EM structure of the *Pse*TnsAB paired-end complex (right end) in the presence of Mg (EMDB-55638) (PDB-9T7L)	Cryo-EM structure of the *Pse*TnsAB paired-end complex (left end) in the presence of Mg (EMDB-57834) (PDB-30JW)	Cryo-EM structure of the *Pse*TnsAB paired-end complex (right end) in the presence of Mn (EMDB-57833) (PDB-30JV)
Data collection and processing			
Magnification	130,000	130,000	130,000
Voltage (kV)	300	300	300
Electron exposure (e^-/Å^2)	62.130	64.201	64.201
Defocus range (μm)	-1.0 to -2.4 (0.2 steps)	-0.8 to -2.6 (0.3 steps)	-0.8 to -2.6 (0.3 steps)
Pixel size (Å)	0.325	0.325	0.325
Symmetry imposed	C2	C2	C2
Initial particle images (no.)	1,095,968	4,935,000	2,242,284
Final particle images (no.)	203,418	575,675	159,536
Map resolution (Å)	2.78	2.51	2.86
FSC threshold	0.143	0.143	0.143
Map resolution range (Å)	2.3-5.0	2.0-4.0	2.5-5.0
Refinement			
Initial model used (PDB code)	AlphaFold 3	9T7L	9T7L
Model resolution (Å)	2.8	2.5	2.8
FSC threshold	0.143	0.143	0.143
Model resolution range (Å)	2.7-3.0	2.5-2.7	2.8-3.1
Map sharpening *B* factor (Å^2)	Unsharpened map	Unsharpened map	Unsharpened map
Model composition<br>Non-hydrogen atoms<br>Protein residues<br>Nucleotide residues<br>Ligands	 17938<br>1,888<br>124<br>MG: 2	 17,940<br>1,888<br>124<br>MG: 2	 17,940<br>1,888<br>124<br>MN: 4
B factors (Å^2) min/max/mean<br>Protein<br>Nucleotide<br>Ligand	 30.00/207.02/132.93<br>80.15/258.18/140.39<br>163.15/163.15/163.15	 59.35/209.03/124.87<br>61.36/255.48/129.51<br>161.53/161.53/161.53	 64.27/216.45/138.28<br>69.14/273.80/145.19<br>160.44/165.24/162.84
R.m.s. deviations<br>Bond lengths (Å)<br>Bond angles (°)	 0.004(0)<br>0.625(0)	 0.003(0)<br>0.602(0)	 0.003(0)<br>0.563(0)
Validation<br>MolProbity score<br>Clashscore<br>Poor rotamers (%)	 1.66<br>6.35<br>2.59	 1.55<br>7.73<br>1.47	 1.68<br>8.08<br>1.94
Ramachandran plot<br>Favored (%)<br>Allowed (%)<br>Disallowed (%)	 98.55<br>1.45<br>0.00	 98.66<br>1.34<br>0.00	 97.90<br>1.99<br>0.11

Supplementary References

1 Park, J. U. *et al.* Structures of the holo CRISPR RNA-guided transposon integration complex. *Nature* **613**, 775-782 (2023). https://doi.org:10.1038/s41586-022-05573-5

2 Wang, S., Siddique, R., Hall, M. C., Rice, P. A. & Chang, L. Structure of TnsABCD transpososome reveals mechanisms of targeted DNA transposition. *Cell* **187**, 6865-6881.e6816 (2024). https://doi.org:https://doi.org/10.1016/j.cell.2024.09.023

3 Kaczmarska, Z. *et al.* Structural basis of transposon end recognition explains central features of Tn7 transposition systems. *Molecular Cell* **82**, 2618-2632.e2617 (2022). https://doi.org:10.1016/j.molcel.2022.05.005

4 Tenjo-Castaño, F. *et al.* Structure of the TnsB transposase-DNA complex of type V-K CRISPR-associated transposon. *Nature Communications* **13**, 5792 (2022). https://doi.org:10.1038/s41467-022-33504-5

5 Abramson, J. *et al.* Accurate structure prediction of biomolecular interactions with AlphaFold 3. *Nature* **630**, 493-500 (2024). https://doi.org:10.1038/s41586-024-07487-w
