# Supplementary material for: Transposon end recognition and excision mechanisms of type I-F CRISPR-associated transposases
Source: Nat Commun. 2026 Aug 24;17:10110. doi: 10.1038/s41467-026-76893-7 (PMC13598128; doi:10.1038/s41467-026-76893-7)
Supplement: Supplementary file 2 [file 41467_2026_76893_MOESM2_ESM.pdf]

Description of Additional Supplementary Files

File name: Supplementary Data 1
Description: Multiple sequence alignment of TnsB proteins from type I-F CASTs. Output from Promals3D[1]; Tn*7016* (*Pseudoalteromonas* sp. strain S983), WP_029772100.1; Tn*6677* (*Vibrio cholerae* strain HE-45), WP_001074608.1; Tn*6900* (*Aeromonas salmonicida*), WP_088821968.1; Tn*7000* (*Vibrio cholerae* strain 4874), WP_119464978.1; Tn*7001* (*Photobacterium iliopiscarium*), WP_107274795.1; Tn*7002* (*Vibrio* sp. strain F12), WP_136985358.1; Tn*7003* (*Vibrio parahaemolyticus*), WP_015313514.1; Tn*7004* (*Vibrio* sp. strain 16), WP_005472985.1; Tn*7005* (*Vibrio cholerae* strain M1517), WP_001134621.1; Tn*7006* (*Vibrio splendidus*), WP_054541713.1; Tn*7008* (*Aliivibrio* sp.), WP_065591601.1; Tn*7011* (*Pseudalteromonas* sp. strain P1-25), WP_055018246.1; Tn*7012* (*Pseudoalteromonas ruthenica*), WP_138590899.1; Tn*7013* (*Vibrio cholerae* strain OYP7G04), WP_114778930.1; Tn*7014* (*Vibrio diazotrophicus*), WP_102952857.1; Tn*7015* (*Shewanella* sp.), WP_076538387.1; Tn*7017* (*Endozoicomonas ascidiicola*), WP_095210281.1[2]. Domain boundaries of *Pse*TnsB are indicated above the alignment; residues described in this study are highlighted in yellow.

File name: Supplementary Data 2
Description: Multiple sequence alignment of TnsA proteins from type I-F CASTs. Output from Promals3D[1]; Tn*7016* (*Pseudoalteromonas* sp. strain S983), WP_029772101.1; Tn*6677* (*Vibrio cholerae* strain HE-45), WP_000202732.1; Tn*6900* (*Aeromonas salmonicida*), WP_088821967.1; Tn*7000* (*Vibrio cholerae* strain 4874), WP_119464977.1; Tn*7001* (*Photobacterium iliopiscarium*), WP_107274794.1; Tn*7002* (*Vibrio* sp. strain F12), WP_136985357.1; Tn*7003* (*Vibrio parahaemolyticus*), WP_015313515.1; Tn*7004* (*Vibrio* sp. strain 16), WP_005472937.1; Tn*7005* (*Vibrio cholerae* strain M1517), WP_000460251.1; Tn*7006* (*Vibrio splendidus*), WP_050622132.1; Tn*7008* (*Aliivibrio* sp.), WP_065591602.1; Tn*7011* (*Pseudalteromonas* sp. strain P1-25), WP_036981971.1; Tn*7012* (*Pseudoalteromonas ruthenica*), WP_138590901.1; Tn*7013* (*Vibrio cholerae* strain OYP7G04), WP_114778920.1; Tn*7014* (*Vibrio diazotrophicus*), WP_102952856.1; Tn*7015* (*Shewanella* sp.), WP_076538389.1; Tn*7017* (*Endozoicomonas ascidiicola*), WP_157673482.1[2]. Domain boundaries of *Pse*TnsA are indicated above the alignment; residues described in this study are highlighted in yellow.

File name: Supplementary Data 3
Description: Description and sequence of oligonucleotides and plasmids used in this study.

File name: Supplementary Data 4
Description: AlphaFold 3 model of evoTnsAB PEC.
