# Supplementary material for: Transposon end recognition and excision mechanisms of type I-F CRISPR-associated transposases
Source: Nat Commun. 2026 Aug 24;17:10110. doi: 10.1038/s41467-026-76893-7 (PMC13598128; doi:10.1038/s41467-026-76893-7)
Supplement: Supplementary file 4 [file 41467_2026_76893_MOESM8_ESM.pdf]

nature portfolio

Peer Review File

Transposon end recognition and excision mechanisms of type I-F CRISPR-associated transposases

Corresponding Author: Dr Irma Querques

This file contains all reviewer reports in order by version, followed by all author rebuttals in order by version.

Version 0:

Reviewer comments:

Reviewer #1

(Remarks to the Author)
CRISPR-associated transposons (CASTs) have the potential to revolutionize genome editing by allowing for programmable RNA-guided kilobase-scale DNA integration. The I-F3 CAST family is particularly attractive due to its high efficiency and its proven effectiveness in human cells. To-date, structural information on the I-F3 family has focused on target-site recognition and has neglected the transposases (TnsAB). Additionally, the molecular determinants of functionality within human cells between I-F orthologs and engineered variants remains unclear.

In this manuscript, Walter, Finocchio, and Oberli et al. present the first high-resolution structure of a post-excision state TnsAB paired-end complex and attempt to determine the molecular features underlying the increased efficiency of the engineered variant evoTnsAB. Furthermore, the authors reveal the sequence-specific recognition of the TnsB binding sites, the mechanism behind transposon end cleavage, PEC assembly, and start to tease apart the coordination between TnsA and TnsB.
Effectively, this manuscript serves as valuable step in understanding the type I-F3 transposases and provides useful information for further optimization of CASTs. Publication in Nature Communications should be contingent on the authors addressing the points below.

1. For Fig 1C, the authors claim that the TnsB+Mn2+ shows minimal nicking activity, however it shows noticeably more relaxed plasmids than the negative control, the TnsAB+Mg2+(the physiological catalytic ion) condition and the TnsAB+Ca2+ condition. Previous studies confirm that TnsA is required to support nucleolytic function, however Fig. 1C does not convincingly support this. The result could be explained as an artifact of the higher Mn2+ concentration used (20mM), which might cast doubt on some of the conclusions in the paper related to cleavage. Additionally, a more general explanation should be added about the role of Mg+ and Mn2+ in their experiments and in nature as it has profound effects on coordination in the EcTn7 system in previous work and is therefore relevant to the new work presented here. The paper is already referenced (PMID: 10704304) but the findings about Mg+ verses Mn2+ is not explained.
2. Supplemental Fig 6 implies that the presence of TnsC does not stimulate TnsAB cleavage activity, however the experiment is performed with high concentrations of Mn2+ which induces a hyperactivity phenotype. This experiment needs to also be performed with the physiological catalytic ion to fully conclude that TnsC does not stimulate TnsAB activity.
3. Line 81 and throughout the paper (Lines 203, 218, 363, 377, 400 and more) the distinctions between the two coopted I-B CAST systems is not indicated leading to confusion in the text. The CRISPR types that were coopted are very different with CAST, but the transposon systems that coopted the CRISPR systems are themselves very different. One is closer to Tn7 (I-B1) but it would be incorrect to say that the I-F system is more distant than the other (I-B2) system. It is also important to point out that in addition to the types of CRISPR systems being very different, in most cases the subtypes of Tn7-like systems are also highly diverged. This works in favor of increasing the importance of their work because it fills in gaps in our understanding of the diverged machinery under the larger umbrella of Tn7 family elements.
4. Line 30: Described as done "…in its post-excision state…" should be clarified that the structure was done on a complex "…assembled in a mock state…" to represent how the structure likely looks in the post-excision state.
5. Line 39: Incorrectly states that transposon TYPICALLY have distinct left and right ends. However, with most transposons there is no specialization of the ends for polarity control. Many transposons have inverted repeats recognized by the transposase, but a very limited number of types have adaptation that allow them to control the orientation of integration.
6. Line 41: Should be corrected to say "most DNA transposons move via..."
7. Line 48: Use of the word "subsequently" is confusing. Breaking and joining are coordinated in the process. This phrase

suggests distinct chemical processes.
8. Line 52 (and in abstract): the I-F3 CAST system is described as “nuclease-deficient”, while technically true at one level, the wording is confusing. The nuclease function is missing because that protein is absent, not that the nuclease activity was made deficient.
9. Paragraph 1-2: For clarity purposes, TnsD being a TniQ family protein should be mentioned.
10. Line 126: Language should be softened here. Manganese clearly alters the cleavage activity if the system does not work without it in vitro. It would be more accurate to say it does not alter its cleavage pattern.
11. Line 329: A supplemental figure should be added so that the readers can understand how well the density fits to the catalytic residues and the Mg2+ ion. The distances between the catalytic residues and the Mg2+ ion should be provided in the figure.
12. Line 339-342: Labeling the distances mentioned in this text might help the reader understand how the triad is not positioned properly for cleavage. Additionally labeling the terminal DNA would help.
13. Line 345: In Supplemental Fig 9 the active site needs to be labelled for clarity purposes.
14. Lines 389-391: “…possibly by TnsC assembled at the target site.” Should include more details about the role of TnsC in the EcTn7 transposase complex. Previous structure work indicates that TnsC forms part of the DNA binding domain of TnsA (PMID: 15257292) and shown to have large interact interfaces (PMID: 10880457). PMID: 10880457 also indicates a previous transposase gain-of-activity which is relevant to the paper and should be explained.

(Remarks on code availability)

Reviewer #2

(Remarks to the Author)
In their study, Walter, Finocchio, and Oberli et al. present the post-excision–state structure and biochemical analysis of the Pseudoalteromonas type I-F CAST transposase PseTnsAB, with TnsA captured in its active conformation. In contrast to other CAST systems, PseCAST functions robustly in human cells. The authors also present data on the enhanced activity of an evolved TnsAB variant, thereby providing insights not only into the natural system but also into an engineered variant. As such, this study will be of significant interest to the CRISPR biology, genome editing, and protein engineering fields, as it advances the mechanistic understanding of type I-F CASTs and provides insights into the molecular mechanism of an engineered variant.

The structural and biochemical data are solid, well analyzed, and appropriately interpreted, and they largely support the authors’ conclusions. The methodology is sound, and the documentation facilitates reproducibility. Overall, I support publication of this well-written manuscript. To further strengthen the mechanistic interpretations derived from the in vitro and in vivo assays on the evolved TnsAB variant, the authors may want to consider determining the cryo-EM structure of the engineered variant.

Major comments:

- A major motivation for this mechanistic study is the application of CASTs in genome editing. However, the mechanistic insights into the enhanced activity of the evolved TnsAB variant are currently limited to few experiments. An experimental structure of the engineered variant would strengthen the interpretations and help provide “a mechanistic foundation for engineering CRISPR-associated transposases”. If structure determination is not feasible, the authors should provide a justification.

Minor comments and suggestions:

- To increase the precision of the cryo-EM model in relevant areas, consider improving relevant cryo-EM map segments through local refinements, for example in the helical, nuclease, and CAT domains surrounding one of the TnsA active sites.

- Line 772, caption of Fig. 1b: the annotation of repeats as squares and spacers as diamonds should be inverted. In the CRISPR schematic, the flanking repeats are shown as diamonds, whereas the squares indicate spacers.

- Fig. 2b: consider annotating amino acids 1–12 and 794–851, and indicate whether these regions are resolved or unresolved.

- Supplementary Fig. 3b: to improve accessibility, consider indicating in the caption that TnsAB and TnsB data are shown on the left and right, respectively.

- Fig. 2e: consider labeling the DNA segments TR, BS1, and BS2.

- Line 212: when interpreting specific interactions (e.g., salt bridges), consider showing the cryo-EM density around the relevant residues and indicating interatomic distances in Figures.

- Line 259: please check the figure panel reference (Fig. 4a or Fig. 4b).

- Supplementary Fig. 8a and Fig. 5b: please show the cryo-EM density around the bound nucleic acids, cofactors, and interacting residues.

- Consider mentioning the recent discovery of novel transposon-associated systems (Makarova et al., DOI: 10.1038/s41564-025-02180-8).

(Remarks on code availability)

Reviewer #3

(Remarks to the Author)
The authors report a high-resolution cryo-EM structure of a type I-F CAST TnsAB paired-end complex assembled on a minimal right-end (RE) excision-product substrate. The structural work is technically solid and the reconstruction quality is high. However, several aspects of the structural interpretation rely on a highly simplified substrate and reaction conditions that may not be fully physiological, and in some cases the conclusions appear to extend beyond what is directly supported by the data. These issues do not invalidate the structural results, but they do affect the strength and generality of some of the mechanistic interpretations.

Mayor points:

- The structure is determined using a short, truncated right-end (RE) excision-product mimic. While the use of a minimal substrate is understandable to reduce heterogeneity and enable high-resolution reconstruction, this choice has important mechanistic implications that would benefit from further discussion.

In this system, the RE alone appears to be less active than reactions containing a mixture of left and right ends, in contrast to other transposition systems. Therefore, the structure may not fully reflect the physiological paired-end complex. Notably, the TnsB catalytic site is captured in an inactive configuration, raising the possibility that the observed state corresponds to a sub-optimal or partially engaged assembly rather than a fully competent excision complex.

The authors should more explicitly acknowledge the limitations imposed by the minimal RE substrate and moderate claims that this structure represents a fully functional PEC. At minimum, the discussion should clarify that the structure likely reflects one specific assembly state rather than the complete physiological spectrum of paired-end configurations.

- The manuscript does not clearly explain why this particular short substrate was chosen, nor does it discuss what is known about the minimum DNA length required for efficient transposition in this system, either in vitro or in vivo. A brief discussion placing the chosen substrate in the context of known functional requirements would help strengthen the structural interpretation.

- A key mechanistic point is that excision activity is observed only in the presence of Mn^{2+}, whereas no activity is detected with Mg^{2+}, even though the cryo-EM structure is determined under Mg^{2+} conditions. Mn^{2+} is well known to relax catalytic constraints in DDE/D-family enzymes, and activity restricted to Mn^{2+} conditions may indicate that additional requirements are needed to support catalysis under physiological Mg^{2+} conditions.

This distinction is particularly relevant given that the TnsB active site is captured in an inactive configuration in the Mg^{2+}-bound structure. The authors should discuss whether the observed conformation reflects a Mg^{2+}-compatible but catalytically inactive assembly state, and how this relates to the Mn^{2+}-dependent activity observed in functional assays. It would also be useful to comment on whether additional protein or DNA components might be required to promote productive excision under Mg^{2+} conditions.

- The authors conclude that TnsC does not influence excision activity. However, the data presented appear to be limited to Mn^{2+} conditions. It is therefore unclear whether TnsC might play a role specifically under Mg^{2+} conditions, where excision is otherwise not detected.

- Mass photometry and SEC data indicate that even in the presence of DNA, a substantial fraction of TnsAB remains monomeric. It is therefore unclear why the authors did not further purify the assembled complex (e.g. by preparative SEC) prior to cryo-EM grid preparation.

While the final reconstruction demonstrates that a homogeneous subset of particles was successfully isolated computationally, clarification is needed as to whether additional biochemical purification was attempted and whether this might have improved sample homogeneity or reduced the need for extensive particle pruning.

Minor points:

- It would be useful to include an angular distribution plot and a representative raw micrograph in Supp Fig 4 to better document data quality and particle orientation coverage.

- An additional supplementary figure showing the quality of fit between the protein and DNA model and the EM density in different regions (e.g., TR, active sites, DNA binding areas) would strengthen confidence in the atomic interpretation.

- In methods that authors state that the micrographs were collected in a Gatan K3 camera, whereas the PDB validation specifies Falcon III.

(Remarks on code availability)

Version 1:

Reviewer comments:

Reviewer #1

(Remarks to the Author)
The revised manuscript addresses all of my concerns from the previous version

(Remarks on code availability)

Reviewer #2

(Remarks to the Author)
The authors have addressed all major comments, and I support publication of the manuscript provided that a few minor issues are addressed:

1.) To provide a structural framework for the evoTnsAB paired-end complex, Walter, Finocchio, and Oberli present AlphaFold 3 models of the excision product/mimicking state. While AF3 is, to a limited extent, capable of predicting nucleic acid–protein structures, such models should be interpreted with caution. To facilitate assessment of the predicted model, it would be good practice to display the structure colored by pLDDT, report the ipTM/pTM values, and include the predicted aligned error (PAE) plot.
In addition, I encourage the authors to reconsider the phrasing in lines 351–353, as AlphaFold is not well suited to predicting the impact of single amino acid substitutions. Although less so than AF2, AF3 still relies on MSAs for structure prediction. As a result, the predicted structures remain effectively constrained by WT sequence information and are therefore expected to reproduce WT coordinates.

2.) The authors have added supplementary figures showing the cryo-EM map fit for relevant side chains. However, for several of the displayed side chains, the map is not clearly visible at the chosen contour level. For example, in Fig. S6a, the proposed interaction between K52 and E315 is not sufficiently supported by the map. Likewise, in Fig. S6b, it is difficult to assess whether the conformations of the R462 and D454 side chains are supported by the density. If the side chains are not well resolved, the authors should state that the residues are in close proximity and may form contacts, rather than assigning specific interactions, as highlighted by dashed lines.
Please also note that some side chains may be mislabeled in the figures; for example, in Fig. S6c, the label for T380 appears to point toward S379.

(Remarks on code availability)

Reviewer #3

(Remarks to the Author)
The revised manuscript has satisfactorily addressed my previous concerns. The additional biochemical experiments, the Mn^{2+}-bound and LE-bound structures, and the expanded discussion substantially strengthen the manuscript. While some mechanistic interpretations remain necessarily speculative given that the structures were determined using minimal post-excision DNA substrates and capture a catalytically inactive TnsB active site, I consider these limitations to be adequately acknowledged and do not believe additional experiments are required.

(Remarks on code availability)

Reviewer #4

(Remarks to the Author)
I co-reviewed this manuscript with one of the reviewers who provided the listed reports. This is part of the Nature Communications initiative to facilitate training in peer review and to provide appropriate recognition for Early Career

Researchers who co-review manuscripts.

(Remarks on code availability)

Open Access This Peer Review File is licensed under a Creative Commons Attribution 4.0 International License, which permits use, sharing, adaptation, distribution and reproduction in any medium or format, as long as you give appropriate credit to the original author(s) and the source, provide a link to the Creative Commons license, and indicate if changes were made.
In cases where reviewers are anonymous, credit should be given to 'Anonymous Referee' and the source.
The images or other third party material in this Peer Review File are included in the article's Creative Commons license, unless indicated otherwise in a credit line to the material. If material is not included in the article's Creative Commons license and your intended use is not permitted by statutory regulation or exceeds the permitted use, you will need to obtain permission directly from the copyright holder.
To view a copy of this license, visit https://creativecommons.org/licenses/by/4.0/

Manuscript NCOMMS-25-102478
Transposon end recognition and excision mechanisms of type I-F CRISPR-associated transposases

Response to Reviewers' comments
We thank the Reviewers for their constructive feedback. We have carefully revised the manuscript to address all comments, and provide a detailed point-by-point response below. We appreciate the valuable input, which has helped improve the clarity and rigor of our study, and hope that the revised manuscript meets the expected standards for publication.

Reviewer #1
Remarks to the authors:

CRISPR-associated transposons (CASTs) have the potential to revolutionize genome editing by allowing for programmable RNA-guided kilobase-scale DNA integration. The I-F3 CAST family is particularly attractive due to its high efficiency and its proven effectiveness in human cells. To-date, structural information on the I-F3 family has focused on target-site recognition and has neglected the transposases (TnsAB). Additionally, the molecular determinants of functionality within human cells between I-F orthologs and engineered variants remains unclear.

In this manuscript, Walter, Finocchio, and Oberli et al. present the first high-resolution structure of a post-excision state TnsAB paired-end complex and attempt to determine the molecular features underlying the increased efficiency of the engineered variant evoTnsAB. Furthermore, the authors reveal the sequence-specific recognition of the TnsB binding sites, the mechanism behind transposon end cleavage, PEC assembly, and start to tease apart the coordination between TnsA and TnsB. Effectively, this manuscript serves as valuable step in understanding the type I-F3 transposases and provides useful information for further optimization of CASTs. Publication in Nature Communications should be contingent on the authors addressing the points below.

We sincerely thank the Reviewer for the positive evaluation of our work, and the recognition of the significance of structural insights into type I CAST transposases and the potential impact of these findings on genome engineering applications. We are grateful for the constructive and detailed comments. Below, we address each point in detail.

1. For Fig 1C, the authors claim that the TnsB+Mn2+ shows minimal nicking activity, however it shows noticeably more relaxed plasmids than the negative control, the TnsAB+Mg2+(the physiological catalytic ion) condition and the TnsAB+Ca2+ condition. Previous studies confirm that TnsA is required to support nucleolytic function, however Fig. 1C does not convincingly support this. The result could be explained as an artifact of the higher Mn2+ concentration used (20mM), which might cast doubt on some of the conclusions in the paper related to cleavage. Additionally, a more general explanation should be added about the role of Mg+ and Mn2+ in their experiments and in nature as it has profound effects on coordination in the EcTn7 system in previous work and is therefore relevant to the new work

presented here. The paper is already referenced (PMID: 10704304) but the findings about Mg+ verses Mn2+ is not explained.

We thank the Reviewer for raising this important point regarding TnsB nicking activity. We agree that, under Mn^{+2} conditions, a higher level of relaxed plasmid is observed compared to the negative control, as shown in Fig. 1c. In the previous manuscript version, we described this activity qualitatively as “minimal”. We have now included a quantitative analysis of these assays in Supplementary Fig. 2b. In addition to the excision rates previously shown (left panel, quantification of excision product), we now also quantify total DNA processing (right panel, including all processed DNA species such as relaxed plasmid). This analysis shows that processing activity by TnsB alone is detectable under Mn^{+2} conditions, as correctly noted by the Reviewer, but is reduced by approximately 3.8-fold as compared to TnsAB. These data support the conclusion that TnsB exhibits limited processing activity in isolation, whereas efficient and coordinated cleavage requires the presence of TnsA. We have revised the Results section accordingly to reflect this quantitative comparison.

Lines 124-128

*‘In the absence of PseTnsA, PseTnsB (**Supplementary Fig. 1a**) exhibited processing activity that was reduced ~3.8-fold compared to PseTnsAB (**Fig. 1c**, **Supplementary Fig. 2b**), indicating that the TnsA subunit is required to support efficient nucleolytic function of TnsB in type I-F CASTs, in analogy with EcTn7[6,8,60].’*

To address the Reviewer’s concern that the observed activity might arise from the relatively high Mn^{2+} concentration (20 mM), we performed excision assays with both TnsAB and TnsB across a range of concentrations (5-20 mM), testing both Mn^{2+} (a, top panel in the figure below) and Mg^{2+} (b, lower panel). Across this concentration range, we did not observe a significant effect of cation concentration on DNA processing activity for either enzyme condition. As the titration analyses did not alter the observed activity trends, we decided not to include the additional data in the manuscript.

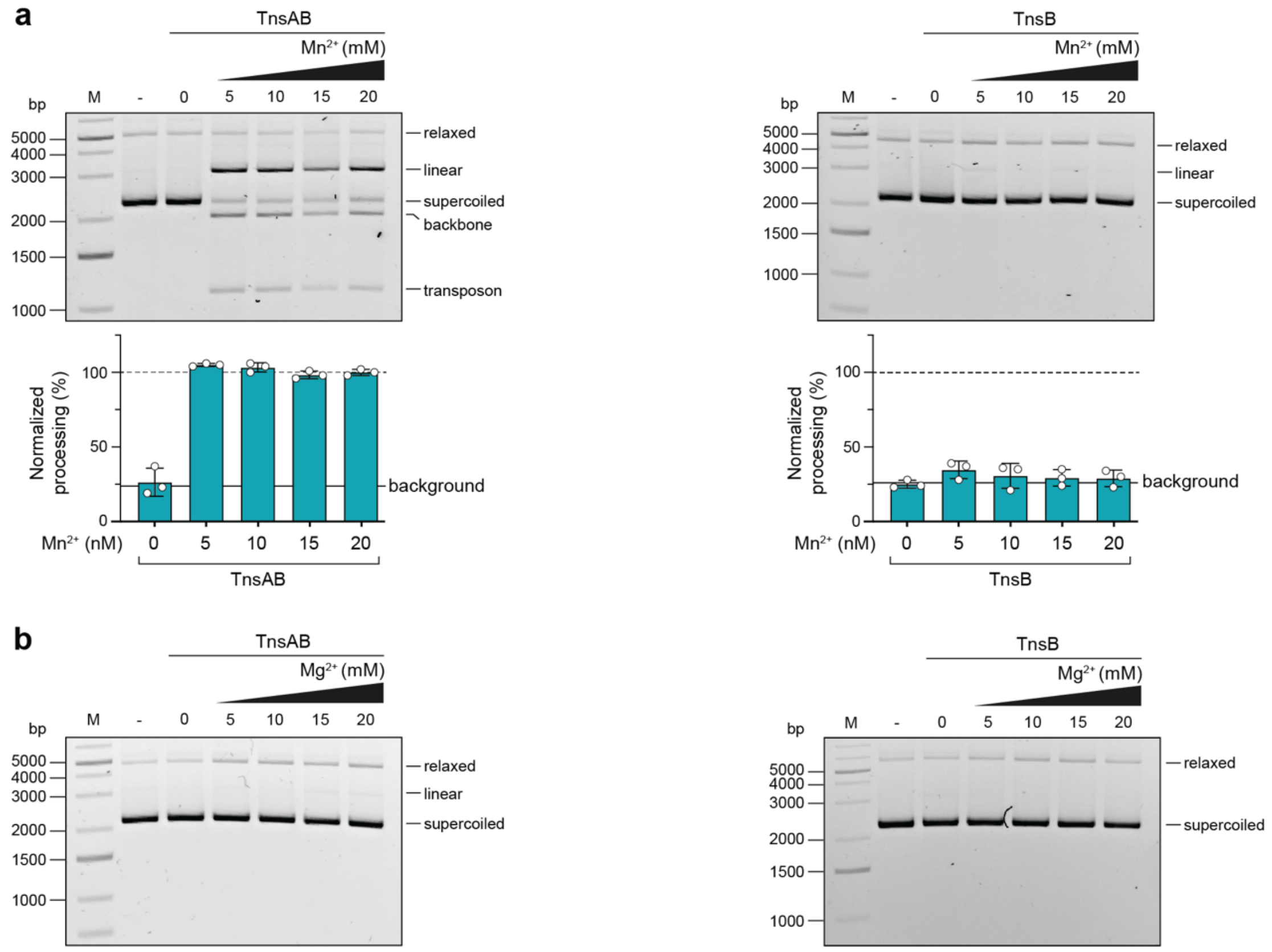

We would also note that we selected the concentration of 20 mM divalent cation for consistency across experiments, including those involving TnsC (see below). This choice was also guided by prior biochemical studies of the *Ec*Tn7 system. For example, reactions in Sarnovsky et al. (EMBO J., 1996) were performed with 15 mM Mg^{2+} in the presence of TnsC, and Biery et al. (J. Mol. Biol., 2000; PMID: 10704304) tested TnsA/TnsB activity across 1.5-15 mM Mn^{2+} or Mg^{2+}. We have clarified this point in the Methods section:

Lines 734-735

'The divalent ion concentrations were chosen based on prior biochemical studies of EcTn7[6,59]*.'*

We agree that clarifying the role of divalent cations in the *Ec*Tn7 system is important for interpreting our findings in *Pse*CAST. To address this, we have expanded the Results section as follows:

Lines 117-124

'In reactions supplemented with Mn^{2+}*, known to relax the catalytic constraints of DDE/D enzymes in vitro*[56]*, we observed product fragments corresponding to a fully excised transposon and a ~2 kb linearized plasmid backbone, along with linear intermediates corresponding to single-end cleavage events (**Fig. 1c**, **Supplementary Fig. 2b**). Such cleavage products were undetectable in the presence of* Mg^{2+}*, the physiological catalytic ion, and with* Ca^{2+}*, which is known to support assembly of cleavage-incompetent transposase complexes*[57,58]*.* Mn^{2+}*-dependent activity is consistent with previous*

biochemical studies of the canonical EcTn7 transposon, where Mn^{2+} promotes cleavage by TnsA and TnsB alone, whereas Mg^{2+} supports catalysis only in the presence of TnsC and targeting factors[6,59].'

2. Supplemental Fig 6 implies that the presence of TnsC does not stimulate TnsAB cleavage activity, however the experiment is performed with high concentrations of Mn2+ which induces a hyperactivity phenotype. This experiment needs to also be performed with the physiological catalytic ion to fully conclude that TnsC does not stimulate TnsAB activity.

We thank the Reviewer for raising this important point. We repeated the experiment in the presence of 20 mM Mg^{2+} under otherwise comparable conditions (Supplementary Fig. 7c in the revised version). Under these conditions, no excised transposon product was detectable upon addition of TnsC, as shown in the representative agarose gel. These data are consistent with our conclusion that, under the assay conditions used in the study, TnsC does not stimulate TnsAB-mediated excision. For consistency with the analysis shown in Supplementary Fig. 2b, we also included quantification of overall DNA processing efficiency under both Mg^{2+} and Mn^{2+} conditions (Supplementary Fig. 7b-c).

The purpose of these experiments was to test whether TnsC directly enhances TnsA-TnsB coordinated cleavage activity through protein-protein interactions. Similar assays have been reported for *Ec*Tn7 (Ronning et al., EMBO J, 2004; PMID: 15257292), where a C-terminal fragment of TnsC was sufficient to stimulate TnsA-TnsB-mediated excision *in vitro* in both Mg^{2+} and Mn^{2+}. In contrast, no stimulation is observed under comparable conditions for the PseCAST TnsAB and TnsC. Importantly, our assays were performed in the absence of ATP and therefore probe excision in the presence of monomeric TnsC. We conceptualized these experiments in light of previous findings on *Ec*Tn7 as follows in the results:

Lines 253-257:

'In EcTn7, the interaction between TnsA and TnsB is mediated in part by TnsC, which forms extensive interfaces with both proteins and is required for transposase activity under physiological Mg^{2+} conditions[58,65]. The crystal structure of TnsA in complex with the C-terminal tail of TnsC reveals an interaction with a hydrophobic patch on the surface of TnsA, which enhances DNA binding and stimulates TnsAB-mediated excision[66].'

3. Line 81 and throughout the paper (Lines 203, 218, 363, 377, 400 and more) the distinctions between the two coopted I-B CAST systems is not indicated leading to confusion in the text. The CRISPR types that were coopted are very different with CAST, but the transposon systems that coopted the CRISPR systems are themselves very different. One is closer to Tn7 (I-B1) but it would be incorrect to say that the I-F system is more distant than the other (I-B2) system. It is also important to point out that in addition to the types of CRISPR systems being very different, in most cases the subtypes of Tn7-like systems are also highly diverged. This works in favor of increasing the importance of their work because

it fills in gaps in our understanding of the diverged machinery under the larger umbrella of Tn7 family elements.

We thank the Reviewer for highlighting these important aspects and clarifications. We have now consistently referred to *Pmc*CAST as a type I-B2 CAST throughout the text and figures. In the Introduction, we explicitly distinguish between the I-B1 and I-B2 systems and note that both the CRISPR systems and the Tns(A)BC machineries are often highly diverged across CAST subtypes.

Lines 70-72:

'CASTs are further subdivided into subtypes, including I-F[17], I-B (I-B1 and I-B2)[19], I-C[33], I-D[20], I-E[34], and V-K[18], with both their CRISPR and Tns(A)BC machineries being highly divergent[33,35].'

We have also removed the previous incorrect statement about the relative phylogenetic relationships between type I-B, *Ec*Tn7, and type I-F CASTs and replaced it with a sentence that reflects the structural knowledge gap.

Lines 88-89:

'The lack of structural information on transposase architecture has limited our mechanistic understanding of type I-F CASTs.'

4. Line 30: Described as done "…in its post-excision state…" should be clarified that the structure was done on a complex "…assembled in a mock state…" to represent how the structure likely looks in the post-excision state.

We thank the Reviewer for this helpful comment. We have improved clarity by removing the phrase "post-excision state" from the Abstract (line 30) and instead making the nature of the structural assembly explicit in the Introduction. We now state that:

Lines 98-101

'*Using cryogenic electron microscopy (cryo-EM), we determined a set of structures of the heteromeric TnsA-TnsB transposase assembled on DNAs mimicking cleaved transposon ends, thus representing the post-excision product state.*'

We have also revised the phrasing in the Results section (lines 166-167) to reflect this point, describing the obtained reconstruction as representing

Lines 180-182

'… a tetrameric PseTnsAB PEC assembled on DNA mimicking the cleaved transposon RE, i.e. in a post-excision, product-bound configuration …'

5. Line 39: Incorrectly states that transposon TYPICALLY have distinct left and right ends. However, with most transposons there is no specialization of the ends for polarity control. Many transposons have inverted repeats recognized by the transposase, but a very limited number of types have adaptation that allow them to control the orientation of integration.

We have corrected the text by removing the reference to distinct LE and RE ends as a general feature of DNA transposons.

Lines 39-42
'*DNA transposons are typically demarcated by end sequences containing inverted repeat motifs that are recognized and cleaved by transposase enzymes during the transposition process*.'

6. Line 41: Should be corrected to say "most DNA transposons move via..."

We have corrected the sentence (line 42 in the revised manuscript).

7. Line 48: Use of the word "subsequently" is confusing. Breaking and joining are coordinated in the process. This phrase suggests distinct chemical processes.

We have removed the word 'subsequently' (now line 50).

8. Line 52 (and in abstract): the I-F3 CAST system is described as "nuclease-deficient", while technically true at one level, the wording is confusing. The nuclease function is missing because that protein is absent, not that the nuclease activity was made deficient.

We have revised the Abstract (lines 23-24) and Introduction (lines 55-56), where we describe these systems as nuclease-less or catalytically inactive CRISPR effectors. This terminology covers both Type I systems, which lack Cas3, and Type V systems, where Cas12k is a naturally occurring pseudonuclease.

9. Paragraph 1-2: For clarity purposes, TnsD being a TniQ family protein should be mentioned.

Thank you for the suggestion. We have now clarified in the Introduction that TnsD belongs to the TniQ protein family (line 53).

10. Line 126: Language should be softened here. Manganese clearly alters the cleavage activity if the system does not work without it in vitro. It would be more accurate to say it does not alter its cleavage pattern.

We agree with the Reviewer. We have adjusted the text as the Reviewer suggested in the revised manuscript (now line 137-139).
'*These findings indicate that artificial fusion of the transposase subunits and the use of manganese as a cofactor do not alter the cleavage pattern of the type I-F CAST transposase.*'

11. Line 329: A supplemental figure should be added so that the readers can understand how well the density fits to the catalytic residues and the Mg2+ ion. The distances between the catalytic residues and the Mg2+ ion should be provided in the figure.

We have added Supplementary Fig. 13b-c to support the statement. See also the detailed description of related edits in the response below.

12. Line 339-342: Labeling the distances mentioned in this text might help the reader understand how the triad is not positioned properly for cleavage. Additionally labeling the terminal DNA would help.

We thank the Reviewer for the helpful suggestions in comments 11 and 12. We have added distance measurements between the catalytic residues and metal ions directly to Fig. 5 and included a new supplementary figure showing the map-to-model fit of the catalytic sites of both TnsA and TnsB, with the atomic model overlaid with the cryo-EM density (Supplementary Fig. 13).

In addition, we have determined structures of the TnsAB paired-end complex bound to transposon right end (RE) DNA in the presence of Mn^{2+}, and to transposon left end (LE) DNA in the presence of Mg^{2+}. These structures provide further insights into metal coordination in the TnsB active site and allow us to assess whether key structural features are conserved in the LE-bound complexes. The corresponding detailed views of the active sites have been incorporated into Fig. 5 and Supplementary Fig. 13. The relevant inter-residue and coordination distances are now visualized in the updated figures, and the text has been revised accordingly. More generally, we now indicate all interaction distances ≤ 3.5 Å as dashed lines in Fig. 3, Fig. 4, Supplementary Figs. 7, 8, 10 and, where appropriate, overlay the cryo-EM density (Supplementary Figs. 6, 7, 8, 13) to facilitate comparison with the interactions described in the text.

13. Line 345: In Supplemental Fig 9 the active site needs to be labelled for clarity purposes.

We thank the Reviewer for this helpful suggestion. The active site has now been clearly labeled in Supplementary Fig. 14f (previously Supplemental Fig 9).

14. Lines 389-391: "…possibly by TnsC assembled at the target site." Should include more details about the role of TnsC in the EcTn7 transposase complex. Previous structure work indicates that TnsC forms part of the DNA binding domain of TnsA (PMID: 15257292) and shown to have large interact interfaces (PMID: 10880457). PMID: 10880457 also indicates a previous transposase gain-of-activity which is relevant to the paper and should be explained.

We thank the Reviewer for this important comment. To better contextualize our model and clarify the role of TnsC, we have expanded the Introduction and Discussion to more explicitly incorporate previous findings from the *Ec*Tn7 system, including the studies suggested by the Reviewer. In particular, we now describe the role of *Ec*TnsC in regulating *Ec*TnsA and *Ec*TnsB activity and its extensive protein-protein interaction interfaces, thereby providing a clearer framework for interpreting our results. We also explicitly highlight that the mode of interaction proposed for *Ec*Tn7 differs from that observed in the structure of the type I-B2 *Pmc*CAST TnsABCD transpososome, underscoring the diversity of TnsC-transposase interactions across systems.

Introduction, lines 253-259

'In EcTn7, the interaction between TnsA and TnsB is mediated in part by TnsC, which forms extensive interfaces with both proteins and is required for transposase activity under physiological Mg^{2+} conditions[58,65]. The crystal structure of TnsA in complex with the C-terminal tail of TnsC reveals an interaction with a hydrophobic patch on the surface of TnsA, which enhances DNA binding and stimulates TnsAB-mediated excision[66]. In the type I-B2 PmcCAST, the interaction between TnsA and TnsB is stabilized by the conserved Arg369-RxxxRD-Asp374 motif in the TnsC C-terminal tail, although the mode of interaction differs from that observed in EcTn7[54].'

Our structural and biochemical data indicate that in the type I-F *Pse*CAST, TnsC is not required to position TnsA for DNA binding and cleavage, whereas TnsB activation may require requires additional steps and formation of transpososome intermediates not captured in our post-excision mimicking structures. In the revised Discussion, we clarify our model in which TnsB activation may depend on donor DNA engagement and target-site capture.

Lines 473-475

'It is conceivable that productive excision under physiological conditions requires engagement of donor DNA by the transposase and binding to target DNA upon recruitment by ATP-bound TnsC oligomers.'

Experimental validation of this model will require reconstitution and structural determination of the full *Pse*CAST transpososome holocomplex, which is ongoing but beyond the scope of the present study.

To further address the Reviewer's point, we now also discuss previously reported gain-of-function mutations in *Ec*Tn7 TnsA-TnsB that enable transposition in the absence of TnsC, likely by stabilizing an active conformation of the transposase. We note that none of these substitutions correspond to mutations present in evoTnsAB. Consistent with this, untargeted integration by evoTnsAB remains TnsC-dependent, indicating that the observed off-target insertions (now described in the Introduction, lines 95) arise from aberrantly assembled TnsABC complexes.

Lines 491-494

‘Of note, none of the substitutions present in evoTnsAB correspond to TnsC-bypassing mutations identified in EcTn7[65]*, suggesting that the observed hyperactivity arises through a distinct mechanism. Consistently, evoTnsAB-mediated off-target insertions in human cells remain dependent on TnsC*[27]*.’*

Reviewer #2

Remarks to the authors:

In their study, Walter, Finocchio, and Oberli et al. present the post-excision–state structure and biochemical analysis of the Pseudoalteromonas type I-F CAST transposase PseTnsAB, with TnsA captured in its active conformation. In contrast to other CAST systems, PseCAST functions robustly in human cells. The authors also present data on the enhanced activity of an evolved TnsAB variant, thereby providing insights not only into the natural system but also into an engineered variant. As such, this study will be of significant interest to the CRISPR biology, genome editing, and protein engineering fields, as it advances the mechanistic understanding of type I-F CASTs and provides insights into the molecular mechanism of an engineered variant.

The structural and biochemical data are solid, well analyzed, and appropriately interpreted, and they largely support the authors' conclusions. The methodology is sound, and the documentation facilitates reproducibility. Overall, I support publication of this well-written manuscript. To further strengthen the mechanistic interpretations derived from the in vitro and in vivo assays on the evolved TnsAB variant, the authors may want to consider determining the cryo-EM structure of the engineered variant.

We thank the Reviewer for the careful and positive evaluation of our work. We are grateful for the recognition of the significance of our structural and biochemical analyses for understanding type I-F CAST mechanisms and their continued optimization.

Major comments:

- A major motivation for this mechanistic study is the application of CASTs in genome editing. However, the mechanistic insights into the enhanced activity of the evolved TnsAB variant are currently limited to few experiments. An experimental structure of the engineered variant would strengthen the interpretations and help provide "a mechanistic foundation for engineering CRISPR-associated transposases". If structure determination is not feasible, the authors should provide a justification.

We thank the Reviewer for this important comment and agree that understanding the mechanistic basis of the enhanced activity of the evolved TnsAB variant is highly relevant for genome engineering applications.

In this study, we compared evoTnsAB and WT TnsAB biochemically in assays probing PEC assembly, DNA binding, and cleavage, corresponding to the steps accessible within our experimental framework. These data do not indicate a measurable increase in activity at the level of excision, suggesting that the observed hyperactivity is due to other factors and likely arises at later stages such as integration (Discussion, lines 494-496).

To provide a structural framework for interpreting the mutations, we generated AlphaFold3 models of the evoTnsAB paired-end complex in the same excision product-mimicking state. These closely match the experimentally determined WT structure (r.m.s.d. 0.885 Å over 374 pruned atom pairs) and are now presented in Supplementary Fig. 11b with all 15 substitutions shown as spheres. Only two substitutions (F43S^{TnsB} and P88T^{TnsA}; highlighted in Supplementary Fig. 11a) map to residues resolved in our structure and could potentially influence DNA binding or stabilization of the TnsA nuclease domain respectively. However, our *in vitro* data do not indicate a measurable increase in activity at these steps. The remaining substitutions are largely surface-exposed or located on hydrophobic patches (e.g., I147V^{TnsA}, V170L^{TnsA}, F180L^{TnsA}, F182L^{TnsA}; A390V^{TnsB}, Q410K^{TnsB}), positioned in flexible loops (e.g., D396N^{TnsB}), or fall within regions not resolved in our reconstruction (e.g., Y349N^{TnsB}, P352T^{TnsB}, V526E^{TnsB}, Q594L^{TnsB}). These features make it difficult to directly attribute their effects to catalytic enhancement in the captured post-excision state and instead suggest possible roles in protein stability, solubility, or other functional steps of the transposition mechanism.

Consistent with this interpretation, previously reported AlphaFold3 models of the PseTnsAB tetramer bound to a DNA substrate mimicking the strand-transfer product (post-integration state) (Witte et al., Science, 2025) suggest that several substitutions may affect DNA binding (e.g., Y349N^{TnsB}) or TnsB-TnsB and TnsB-TnsC interfaces (e.g., P352T^{TnsB}, D396N^{TnsB}, V526E^{TnsB}), which are not visible in our experimentally determined structure. Similarly, substitutions in the C-terminal region (e.g., Q594L^{TnsB}) are predicted to localize at TnsB-TnsC interfaces. These observations indicate that determining a structure of evoTnsAB in the same post-excision state would likely provide limited additional mechanistic insight. Instead, a structurally informative comparison might require capturing a later functional state, such as a target-capture or strand-transfer complex, including TnsC and target DNA. These studies are ongoing but are beyond the scope of the present work.

We have revised the corresponding sections of the manuscript to incorporate these aspects, as follows:

Results, lines 351-353
'*This is consistent with the AlphaFold3 models of the evoCAST TnsAB STC*[27] *and PEC (**Supplementary Fig. 11b**), which closely match the experimental structure (r.m.s.d. 0.885 Å over 374 pruned atom pairs).*'

Finally, to further contextualize the evolved PseTnsAB variant, we examined previously reported gain-of-function mutations in *Ec*Tn7 TnsA-TnsB that increase insertion rates and, in some cases, enable transposition in the absence of TnsC. Notably, none of the corresponding substitutions are present in evoTnsAB, and off-target integration by evoTnsAB remains TnsC-dependent, supporting a distinct mechanistic basis for the observed hyperactivity and off-target insertions in human cells (now described in the Introduction, lines 95-97).

Lines 491-494

'Of note, none of the substitutions present in evoTnsAB correspond to TnsC-bypassing mutations identified in EcTn7[65], suggesting that the observed hyperactivity arises through a distinct mechanism. Consistently, evoTnsAB-mediated off-target insertions in human cells remain dependent on TnsC[27].'

Minor comments and suggestions:

- To increase the precision of the cryo-EM model in relevant areas, consider improving relevant cryo-EM map segments through local refinements, for example in the helical, nuclease, and CAT domains surrounding one of the TnsA active sites.

We thank the Reviewer for this suggestion. We extensively explored local refinement approaches. However, we found that reliable alignment requires relatively large masked regions, which limits their applicability to smaller domains such as those surrounding the TnsA active site. We believe that the current reconstruction already provides well-resolved density in these regions, as supported by the model-to-map agreement shown in revised Supplementary Figs. 6, 7, 8, and 13. In addition, we now include an independently reconstructed dataset at higher resolution for the complex assembled on the transposon left end in the presence of Mg^{2+} (2.51 Å; Supplementary Fig. 10 and Table 1), as well as an Mn^{2+}-bound structure at comparable resolution (2.86 Å; Supplementary Figs. 13-14 and Table 1), both of which further support the quality and interpretation of the structural models.

- Line 772, caption of Fig. 1b: the annotation of repeats as squares and spacers as diamonds should be inverted. In the CRISPR schematic, the flanking repeats are shown as diamonds, whereas the squares indicate spacers.

We thank the Reviewer for spotting this inversion. The figure caption has been corrected in the revised manuscript (now line 938).

- Fig. 2b: consider annotating amino acids 1–12 and 794–851, and indicate whether these regions are resolved or unresolved.

We have revised Fig. 2b to clearly indicate which regions of TnsA/TnsB are resolved or unresolved in the structure. Amino acids 1-12 and 794-851 are now explicitly labeled as the N-terminal and C-terminal regions, respectively, and are annotated in the figure as flexible, unresolved termini. This clarification is also included in the updated figure caption.

- Supplementary Fig. 3b: to improve accessibility, consider indicating in the caption that TnsAB and TnsB data are shown on the left and right, respectively.

We have updated the caption of Supplementary Fig. 3b accordingly.

- Fig. 2e: consider labeling the DNA segments TR, BS1, and BS2.

The DNA segments have now been labeled in Fig. 2e for clarity.

- Line 212: when interpreting specific interactions (e.g., salt bridges), consider showing the cryo-EM density around the relevant residues and indicating interatomic distances in Figures.

We thank the Reviewer for this helpful suggestion. To improve clarity while maintaining figure readability, we have included new supplementary figures showing the cryo-EM density as a transparent mesh overlaid with the atomic model for the regions highlighted in the main figures (Supplementary Fig. 6, 7, 8, 13). We have also added distance measurements between the catalytic residues and metal ions directly to Fig. 5 and included a new supplementary figure showing the map-to-model fit of the catalytic sites of both TnsA and TnsB, with the atomic model overlaid with the cryo-EM density (Supplementary Fig. 13).

We find it difficult to include all labels as well as cryo-EM density in the main panel figures without compromising the clarity of the figures. To avoid visual clutter, we now indicate relevant atomic contacts (hydrogen bonds, salt bridges and metal coordination) ≤ 3.5 Å as dashed lines in Fig. 3-5 and overlay cryo-EM density in Supplementary Figs. 6, 7, 8, 13 to facilitate reference to the interactions described in the text.

- Line 259: please check the figure panel reference (Fig. 4a or Fig. 4b).

We are very grateful the Reviewer for spotting this mistake. It has now been corrected to Fig. 4b in the revised document (line 286 in the revised version).

- Supplementary Fig. 8a and Fig. 5b: please show the cryo-EM density around the bound nucleic acids, cofactors, and interacting residues.

This has been addressed in Supplementary Fig. 6, 7, 8, 13 as described in our response above and in the updated figure captions.

- Consider mentioning the recent discovery of novel transposon-associated systems (Makarova et al., DOI: 10.1038/s41564-025-02180-8).

Thank you for the suggestion. We have added a sentence in line 71-72 referring to Makarova et al. 2025 (ref. 33), noting the novel CAST systems identified bioinformatically.

'*CASTs are further subdivided into subtypes, including I-F*[17]*, I-B (I-B1 and I-B2)*[19]*, I-C*[33]*, I-D*[20]*, I-E*[34]*, and V-K*[18]*, with both their CRISPR and Tns(A)BC machineries being highly divergent*[33,35]*.*'

Reviewer #3

Remarks to the authors:

The authors report a high-resolution cryo-EM structure of a type I-F CAST TnsAB paired-end complex assembled on a minimal right-end (RE) excision-product substrate. The structural work is technically solid and the reconstruction quality is high. However, several aspects of the structural interpretation rely on a highly simplified substrate and reaction conditions that may not be fully physiological, and in some cases the conclusions appear to extend beyond what is directly supported by the data. These issues do not invalidate the structural results, but they do affect the strength and generality of some of the mechanistic interpretations.

We thank the Reviewer for the extensive evaluation of our work and for recognizing the technical quality of the cryo-EM analysis. In response to the concerns raised, we have revised the manuscript to clearly acknowledge the limitations of the minimal substrate used for structure determination and to moderate our interpretations accordingly. In addition, we have included new experiments addressing the functional requirements of the RE DNA for complex formation. We have also determined structures of the complex bound to transposon right end (RE) DNA in the presence of Mn^{2+} and to transposon left end (LE) DNA in the presence of Mg^{2+} to provide complementary structural insights.

We believe these additions and revisions appropriately complement the prior structural and biochemical data and strengthen the mechanistic conclusions.

Mayor points:

- The structure is determined using a short, truncated right-end (RE) excision-product mimic. While the use of a minimal substrate is understandable to reduce heterogeneity and enable high-resolution reconstruction, this choice has important mechanistic implications that would benefit from further discussion. In this system, the RE alone appears to be less active than reactions containing a mixture of left and right ends, in contrast to other transposition systems. Therefore, the structure may not fully reflect the physiological paired-end complex. Notably, the TnsB catalytic site is captured in an inactive configuration, raising the possibility that the observed state corresponds to a sub-optimal or partially engaged assembly rather than a fully competent excision complex.
The authors should more explicitly acknowledge the limitations imposed by the minimal RE substrate and moderate claims that this structure represents a fully functional PEC. At minimum, the discussion should clarify that the structure likely reflects one specific assembly state rather than the complete physiological spectrum of paired-end configurations.

- The manuscript does not clearly explain why this particular short substrate was chosen, nor does it discuss what is known about the minimum DNA length required for efficient transposition in this system,

either in vitro or in vivo. A brief discussion placing the chosen substrate in the context of known functional requirements would help strengthen the structural interpretation.

We thank the Reviewer for these insightful comments and address both points jointly. We agree that the use of a minimal RE substrate may limit the extent to which the structure captures the full physiological paired-end complex (PEC), and we have clarified this throughout the revised manuscript and moderated our interpretations accordingly.

The truncated RE substrate was selected to reduce conformational heterogeneity and enable high-resolution reconstruction. In *Pse*CAST, the two outermost TnsB binding sites are located in close proximity at the right end, whereas they are separated by 36 bp at the left end (Supplementary Fig. 2d). Based on previous structural studies indicating that at least two TnsB binding sites are required for transpososome assembly (Tenjo-Castaño et al., 2022; Park et al., 2022; Kaczmarska et al., 2022), we initially focused on a minimal RE substrate comprising BS1 and BS2. The final design, including BS1 and 15 base pairs of BS2, was further guided by prior structural work on the type I-B2 CAST *Pmc*TnsABCD transpososome, in which longer DNA substrates were used, but cryo-EM density was resolved primarily for a single binding site and only a small portion of BS2 (Wang et al., 2024). We have clarified this rationale in the revised manuscript (Results, lines 159-166).

'To gain insights into the molecular mechanisms underpinning transposon end recognition and cleavage in type I-F CASTs, we proceeded to determine a cryo-EM structure of a PseTnsAB paired-end complex (PEC). We initially focused on the RE, as previous studies showed that the presence of a minimum of two closely spaced binding sites is required for complex formation[41,42,54,55]. To facilitate structural analysis and minimize sample heterogeneity, we used a double-stranded DNA mimicking the RE excision product, comprising BS1 and 15 base pairs of BS2. The final design was informed by the structure of the type I-B2 CAST PmcTnsABCD transpososome, in which density was resolved primarily for BS1 and only a portion of BS2[54].'

Prior *in vivo* experiments (Lampe et al., *Nat. Biotechnol.*, 2024, PMID 36991112) demonstrated that three binding sites on both the RE and LE are required for efficient transposition in *E. coli* (lines 143-145 and Supplementary Fig. 2d). Our *in vitro* data show that two sites per end support detectable, albeit reduced, excision (Supplementary Fig. 2g). To further address the Reviewer's point, we now include additional experiments demonstrating that BS1 alone (with BS2 mutated) is sufficient to support RE-RE PEC assembly *in vitro* (new Supplementary Fig. 9), indicating that a single binding site can support end pairing through transposase tetramerization. Consistently, we have also determined a structure of TnsAB bound to a left end (LE) DNA substrate consisting of BS1 alone, which adopts a highly similar overall architecture to the RE-bound complex (Supplementary Fig. 10). The close agreement between these structures suggests that the observed structural organization represents a conserved assembly state likely maintained in LE-RE complexes. These new data are incorporated in the revised Results (lines 313-341).

We have revised the Discussion to clarify that the structure likely represents a defined assembly state rather than the full spectrum of physiological PEC configurations (Discussion, lines 452-455), and we have adjusted terminology, specifically the term “PEC,” throughout the manuscript to avoid overgeneralization and prevent misinterpretation.

Regarding the inactive configuration of the TnsB catalytic site, we note that the DNA substrate used here mimics a post-cleavage product, lacking the downstream donor DNA, and it is therefore plausible that the complex relaxes into a non-productive conformation following cleavage and release of the downstream DNA. Alternatively, the catalytically active configuration may represent a transient, higher-energy state that is not highly populated under the conditions used for structure determination.

Taken together, we interpret the structures as mechanistically relevant assembly states that provide insight into specific recognition of both the right and left transposon ends, end pairing, and subunit organization, while acknowledging that full catalytic activation likely requires additional DNA elements and/or interaction with other factors, including TnsC, under physiological conditions.

- A key mechanistic point is that excision activity is observed only in the presence of Mn^{2+}, whereas no activity is detected with Mg^{2+}, even though the cryo-EM structure is determined under Mg^{2+} conditions. Mn^{2+} is well known to relax catalytic constraints in DDE/D-family enzymes, and activity restricted to Mn^{2+} conditions may indicate that additional requirements are needed to support catalysis under physiological Mg^{2+} conditions.

This distinction is particularly relevant given that the TnsB active site is captured in an inactive configuration in the Mg^{2+}-bound structure. The authors should discuss whether the observed conformation reflects a Mg^{2+}-compatible but catalytically inactive assembly state, and how this relates to the Mn^{2+}-dependent activity observed in functional assays. It would also be useful to comment on whether additional protein or DNA components might be required to promote productive excision under Mg^{2+} conditions.

We thank the Reviewer for bringing up this important point. We agree that the distinction between Mn^{2+}- and Mg^{2+}-dependent activity is critical for interpreting both the structural and biochemical data.

*Pse*TnsAB excision activity restricted to Mn^{2+} conditions likely reflects the ability of this ion to stabilize catalytically permissive conformations that are not readily accessible under physiological Mg^{2+} conditions *in vitro*. This behavior is consistent with previous studies of the canonical *Ec*Tn7 system, where Mn^{2+} supports cleavage by TnsA and TnsB alone, wherease under Mg^{2+} conditions, complete excision requires the presence of TnsC and targeting factors to support catalysis (now specified in the Results, lines 122-124).

To further investigate the structural basis of this difference, we determined an additional cryo-EM structure of the RE-assembled complex in the presence of Mn^{2+} at 2.86 Å resolution (Fig. 5d, Supplementary Figs. 13f and 14a-e, Supplementary Table 1. In contrast to the initially determined structure, where density for the Mg^{2+} ion is not discernible in the TnsB active site, this structure reveals clear density consistent with a Mn^{2+} ion coordinated by $Asp220^{TnsB}$ and $Asp304^{TnsB}$. However, the overall conformation of the catalytic triad is highly similar to that observed in the Mg^{2+}-bound structures, with $Glu340^{TnsB}$ remaining positioned distally and thus incompatible with catalysis These observations suggest that the captured conformation represents a Mn^{2+}-bound but catalytically inactive assembly state and that Mn^{2+} may have a higher binding affinity to TnsB active site than Mg^{2+}, yet the presence of Mn^{2+} alone is not sufficient to remodel the TnsB active site into a catalytically competent configuration. The Mn^{2+}-dependent activity observed *in vitro* may therefore arise from altered metal ion affinity and coordination properties, which could facilitate transient formation of a catalytically competent state that is not highly populated and thus not captured in our structures.

The additional structural analysis is now described in the Results section, lines 401-410.
*'To further investigate the organization of the TnsB active site, we determined a structure of an RE-assembled PEC in the presence of Mn^{2+} at an overall resolution of 2.86 Å (**Fig. 5d**, **Supplementary Fig. 14a-e**, **Supplementary Table 1**). Additional density is consistent with a single Mn^{2+} ion coordinated by $Asp220^{TnsB}$ and $Asp304^{TnsB}$ of the catalytic protomers (**Fig. 5d**, **Supplementary Fig. 13f**). The catalytic triad adopts a similar conformation to that observed in the Mg^{2+}-bound structures and remains incompatible with catalysis, with $Glu340^{TnsB}$ positioned distally. These observations suggest that Mn^{2+} may bind with higher affinity than Mg^{2+}, potentially contributing to enhanced in vitro activity, although further studies are required to determine how Mn^{2+} coordination promotes the formation of a catalytically competent active site conformation under these conditions.'*

Additional factors, such as donor DNA engagement, and/or recruitment by TnsC to the target DNA, may be required to stabilize a fully active, excision-competent conformation under physiological Mg^{2+} conditions, as explicitly stated in the Discussion section, lines 473-475
'It is conceivable that productive excision under physiological conditions requires engagement of donor DNA by the transposase and binding to target DNA upon recruitment by ATP-bound TnsC oligomers.'

We also thank the Reviewer for prompting clarification of the mechanistic conclusions derived from our biochemical and structural data, particularly concerning the proposed model of TnsB activation and the molecular requirements underlying this process. We repeated the relevant biochemical experiments with an increased number of independent replicates (n = 6; Fig. 5e) and revised the corresponding Results section (lines 418-429) accordingly. In addition, we updated the model in Fig. 6 to avoid implying a strict separation between NTS and TS cleavage by TnsA and TnsB, respectively. Instead, the revised model figure reflects the coordinated and interdependent contributions of both subunits to excision, which reflects one of the major findings of our study.

- The authors conclude that TnsC does not influence excision activity. However, the data presented appear to be limited to Mn^{2+} conditions. It is therefore unclear whether TnsC might play a role specifically under Mg^{2+} conditions, where excision is otherwise not detected.

We thank the Reviewer for raising this important point. To address this, we repeated the excision assays in the presence of Mg^{2+} under otherwise comparable conditions (20 mM), including TnsC (Supplementary Fig. 7c in the revised version). Under these conditions, no excised transposon product was detectable upon addition of TnsC, as shown in the representative agarose gel. These data are consistent with our conclusion that, under the experimental conditions used in the study, TnsC does not stimulate TnsAB-mediated excision.

The purpose of these experiments was to test whether TnsC directly enhances TnsA-TnsB coordinated cleavage activity through protein-protein interactions. Similar assays have been reported for *Ec*Tn7 (Ronning et al., EMBO J, 2004; PMID: 15257292), where a C-terminal fragment of TnsC was sufficient to stimulate TnsA-TnsB-mediated excision in vitro in both Mg^{2+} and Mn^{2+}. In contrast, no stimulation is observed under comparable conditions for the PseCAST TnsAB and TnsC. Importantly, our assays were performed in the absence of ATP and therefore probe excision in the presence of monomeric TnsC.

We conceptualized these experiments in light of previous findings on *Ec*Tn7 in the Results section, lines 122-124:
'Mn^{2+}-dependent activity is consistent with previous biochemical studies of the canonical EcTn7 transposon, where Mn^{2+} promotes cleavage by TnsA and TnsB alone, whereas Mg^{2+} supports catalysis only in the presence of TnsC and targeting factors[6,59].'

and 253-257:
'In EcTn7, the interaction between TnsA and TnsB is mediated in part by TnsC, which forms extensive interfaces with both proteins and is required for transposase activity under physiological Mg^{2+} conditions[58,65]. The crystal structure of TnsA in complex with the C-terminal tail of TnsC reveals an interaction with a hydrophobic patch on the surface of TnsA, which enhances DNA binding and stimulates TnsAB-mediated excision[66].'

Together, our data suggest that the lack of Mg^{2+}-dependent activity might reflect the absence of additional DNA components and/or assembly states required for productive catalysis. We have clarified this point in the revised manuscript and explicitly discussed the requirement for higher-order TnsC assemblies and target DNA engagement in enabling catalysis under physiological conditions, as noted above (Discussion lines 473-475).

- Mass photometry and SEC data indicate that even in the presence of DNA, a substantial fraction of

TnsAB remains monomeric. It is therefore unclear why the authors did not further purify the assembled complex (e.g. by preparative SEC) prior to cryo-EM grid preparation.
While the final reconstruction demonstrates that a homogeneous subset of particles was successfully isolated computationally, clarification is needed as to whether additional biochemical purification was attempted and whether this might have improved sample homogeneity or reduced the need for extensive particle pruning.

To address this point, we have now included additional mass photometry and analytical SEC data demonstrating that paired-end complexes (tetramers) can be separated from single-end–bound species (monomers) and independently analyzed (new Supplementary Fig. 9). While these experiments were primarily designed to assess the role of the TR sequence in RE synapsis, they also demonstrate that biochemical separation of distinct assembly states is feasible, as outlined in the Results section, lines 313-322:
*'To further dissect the contribution of transposon end DNA sequences to transpososome assembly, we analyzed complex formation by SEC (**Supplementary Fig. 9a**) using RE DNAs identical to those used for cryo-EM, either with wild-type sequence or with scrambling mutations in BS2 or the TR (**Supplementary Fig. 9b**). Analytical SEC revealed two distinct nucleoprotein assemblies when using wild-type or BS2-mutated RE DNAs. In these cases, the two species were observed at comparable ratios. In contrast, TR-mutated DNAs predominantly yielded the lower-order assembly (**Supplementary Fig. 9c**). Mass photometry analysis confirmed these species as tetrameric TnsAB assembled on two RE DNA molecules and monomeric TnsAB bound to a single RE DNA molecule (**Supplementary Fig. 9d**). These experiments indicate that engagement of a second binding site at the right end is not required for tetramerization and end pairing, whereas an intact TR sequence is critical.'*

For structural analysis, we chose not to perform preparative SEC prior to grid preparation in order to preserve the full spectrum of assembly states and maximize the likelihood of capturing different complexes. Moreover, we reasoned that additional purification could be counterproductive, as the removal of excess solutes might alter the particle environment and negatively impact particle distribution and stability. This is particularly relevant given that *Pse*TnsAB is prone to precipitation under low-salt conditions. For these reasons, we opted for a streamlined sample preparation workflow. Instead, computational classification enabled the isolation of a homogeneous subset of particles corresponding to tetrameric complexes. The resulting reconstructions are of high quality and resolution for all structures analyzed, including the right end-bound complex, the left end-bound complex, and the Mn^{2+}-bound complex, indicating that additional biochemical purification was not required to obtain well-defined structural states.

Minor points:
- It would be useful to include an angular distribution plot and a representative raw micrograph in Supp Fig 4 to better document data quality and particle orientation coverage.

We have added an angular distribution plot and a representative raw micrograph to Supplementary Fig. 4. In addition, the same information is now provided for the left end-bound structure (Supplementary Fig. 10) and the Mn^{2+}-bound structure (Supplementary Fig. 14).

- An additional supplementary figure showing the quality of fit between the protein and DNA model and the EM density in different regions (e.g., TR, active sites, DNA binding areas) would strengthen confidence in the atomic interpretation.

We thank the Reviewer for this helpful suggestion. We have included new supplementary figures showing the cryo-EM density as a transparent mesh overlaid with the atomic model for the regions highlighted in the main figures, including DNA and protein interfaces (Supplementary Fig. 6, 7, 8, 13).

- In methods that authors state that the micrographs were collected in a Gatan K3 camera, whereas the PDB validation specifies Falcon III.

We thank the Reviewer for pointing this out. The micrographs were collected using a Gatan K3 camera, as correctly stated in the Methods. The discrepancy in the PDB validation report has been brought to the attention of the PDB curators and has been corrected accordingly.

Manuscript NCOMMS-25-102478
Transposon end recognition and excision mechanisms of type I-F CRISPR-associated transposases

Response to Reviewers' comments from the second round of review.

Reviewer #1

Remarks to the authors:

The revised manuscript addresses all of my concerns from the previous version.

We sincerely thank the reviewer for the positive evaluation of our revised manuscript.

Reviewer #2

Remarks to the authors:

The authors have addressed all major comments, and I support publication of the manuscript provided that a few minor issues are addressed:

1.) To provide a structural framework for the evoTnsAB paired-end complex, Walter, Finocchio, and Oberli present AlphaFold 3 models of the excision product/mimicking state. While AF3 is, to a limited extent, capable of predicting nucleic acid–protein structures, such models should be interpreted with caution. To facilitate assessment of the predicted model, it would be good practice to display the structure colored by pLDDT, report the ipTM/pTM values, and include the predicted aligned error (PAE) plot.
In addition, I encourage the authors to reconsider the phrasing in lines 351–353, as AlphaFold is not well suited to predicting the impact of single amino acid substitutions. Although less so than AF2, AF3 still relies on MSAs for structure prediction. As a result, the predicted structures remain effectively constrained by WT sequence information and are therefore expected to reproduce WT coordinates.

2.) The authors have added supplementary figures showing the cryo-EM map fit for relevant side chains. However, for several of the displayed side chains, the map is not clearly visible at the chosen contour level. For example, in Fig. S6a, the proposed interaction between K52 and E315 is not sufficiently supported by the map. Likewise, in Fig. S6b, it is difficult to assess whether the conformations of the R462 and D454 side chains are supported by the density. If the side chains are not well resolved, the authors should state that the residues are in close proximity and may form contacts, rather than assigning specific interactions, as highlighted by dashed lines.
Please also note that some side chains may be mislabeled in the figures; for example, in Fig. S6c, the label for T380 appears to point toward S379.

We thank the Reviewer for the careful evaluation of our revised manuscript and for the positive assessment supporting its publication. We appreciate the constructive suggestions and have addressed each of the remaining minor comments below.

1) We agree that AlphaFold 3 is not suitable for inferring the structural consequences of individual amino acid substitutions. We have therefore revised the text to avoid implying that the AF 3 models provide evidence for the effects of the mutations. The revised text now states (lines 345-348): ″For comparison, AlphaFold 3 models of the evoCAST TnsAB STC[27] and PEC (**Supplementary Fig. 11b**-**d**) recapitulate the overall architecture of the complex, with the PEC model closely matching the experimental structure (r.m.s.d. 0.885 Å over 374 pruned atom pairs).″

As suggested by the Reviewer, we have also expanded Supplementary Fig. 11 to facilitate assessment of the predicted model. Specifically, we now show the AlphaFold 3 model coloured by per-residue pLDDT (from two viewing angles), report the corresponding ipTM and pTM confidence scores in the legend, and include the predicted aligned error (PAE) plot.

2) We thank the Reviewer for this suggestion. We have replaced the map-to-model fit panels with versions that provide improved contrast to facilitate assessment of the density. We also swapped panels **c** and **d** in Supplementary Fig. 6 to match the revised panel arrangement in the main Fig. 3 following figure reformatting (see below). In addition, we corrected the misplaced label for T380 in Supplementary Fig. 6d (previously Supplementary Fig. 6c).
Regarding the proposed interactions, we carefully re-evaluated the density for all highlighted residue pairs. In Supplementary Fig. 6b, the density clearly supports the conformations of both R462 and D454 and their proposed interaction. Likewise, all other indicated residue pairs are supported by the available density.
For the K52-E315 interaction in Supplementary Fig. 6a, the density clearly supports E315, whereas K52 is well resolved at the backbone level and its side chain is oriented toward E315 but is not fully resolved. We note that this interaction is more clearly supported in the higher-resolution Mg-LE map. We therefore consider the proposed interaction to be supported by the combined structural evidence and have retained the dashed line in the corresponding main Fig. 3a.

Reviewer #3

Remarks to the authors:

The revised manuscript has satisfactorily addressed my previous concerns. The additional biochemical experiments, the Mn^{2+}-bound and LE-bound structures, and the expanded discussion substantially strengthen the manuscript. While some mechanistic interpretations remain necessarily speculative given that the structures were determined using minimal post-excision DNA substrates and capture a catalytically inactive TnsB active site, I consider these limitations to be adequately acknowledged and do not believe additional experiments are required.

We thank the Reviewer for their careful evaluation of our revised manuscript and for their positive assessment. We are grateful for the Reviewer's constructive comments throughout the review process, which have helped strengthen our study.

Reviewer #4

Remarks to the authors:

I co-reviewed this manuscript with one of the reviewers who provided the listed reports. This is part of the Nature Communications initiative to facilitate training in peer review and to provide appropriate recognition for Early Career Researchers who co-review manuscripts.

We thank the co-Reviewer for their time and thoughtful contribution to the evaluation of our manuscript.
