# Supplementary material for: Transposon end recognition and excision mechanisms of type I-F CRISPR-associated transposases
Source: Nat Commun. 2026 Aug 24;17:10110. doi: 10.1038/s41467-026-76893-7 (PMC13598128; doi:10.1038/s41467-026-76893-7)
Supplement: Supplementary file 6 [file 41467_2026_76893_MOESM4_ESM.docx]

**Supplementary Data 2**

**References**

1	Pei, J., Kim, B. H. & Grishin, N. V. PROMALS3D: a tool for multiple protein sequence and structure alignments. *Nucleic Acids Res* **36**, 2295-2300 (2008). <https://doi.org:10.1093/nar/gkn072>

2	Klompe, S. E., Jaber, N., Beh, L. Y., Mohabir, J. T., Bernheim, A. & Sternberg, S. H. Evolutionary and mechanistic diversity of Type I-F CRISPR-associated transposons. *Mol Cell* **82**, 616-628.e615 (2022). <https://doi.org:10.1016/j.molcel.2021.12.021>
